# Microglial TNFR2 signaling regulates the inflammatory response after CNS injury in a sex-specific fashion

**DOI:** 10.1016/j.bbi.2023.12.025

**Published:** 2023-12-22

**Authors:** Stefano Raffaele, Estrid Thougaard, Cathrine C.H. Laursen, Han Gao, Katrine M. Andersen, Pernille V. Nielsen, Natalia Ortí-Casañ, Morten Blichfeldt-Eckhardt, Simon Koch, Milani Deb-Chatterji, Tim Magnus, Jane Stubbe, Kirsten Madsen, Morten Meyer, Matilda Degn, Ulrich L.M. Eisel, Agnieszka Wlodarczyk, Marta Fumagalli, Bettina H. Clausen, Roberta Brambilla, Kate L. Lambertsen

**Affiliations:** a Department of Neurobiology Research, Institute of Molecular Medicine, University of Southern Denmark, 5230 Odense M, Denmark; b Department of Pharmacological and Biomolecular Sciences “Rodolfo Paoletti”, Università degli Studi di Milano, 20133 Milan, Italy; c BRIDGE-Brain Research Inter Disciplinary Guided Excellence, Department of Clinical Research, University of Southern Denmark, 5230 Odense M, Denmark; d Department of Spine Surgery, The Third Affiliated Hospital of Sun Yat-Sen University, 510630 Guangzhou, China; e Guangdong Provincial Center for Engineering and Technology Research of Minimally Invasive Spine Surgery, 510630 Guangzhou, China; f Department of Molecular Neurobiology, Groningen Institute for Evolutionary Life Sciences, University of Groningen, Groningen 9713 AV, Netherlands; g Department of Anaesthesiology, Vejle Hospital, 7100 Vejle, Denmark; h Department of Clinical Research, University of Southern Denmark, 5230 Odense M, Denmark; i Department of Neurology, University Medical Center Hamburg-Eppendorf, 20246 Hamburg, Germany; j Department of Cardiovascular and Renal Research, Institute of Molecular Medicine, University of Southern Denmark, 5230 Odense M, Denmark; k Department of Neurology, Odense University Hospital, 5000 Odense C, Denmark; l Gubra, 2970 Hørsholm, Denmark; m The Miami Project to Cure Paralysis, University of Miami Miller School of Medicine, Miami FL, USA

**Keywords:** Microglia, TNFR2, Stroke, Spinal cord injury, Neuroinflammation

## Abstract

Microglia, the resident immune cells of the central nervous system (CNS), play a major role in damage progression and tissue remodeling after acute CNS injury, including ischemic stroke (IS) and spinal cord injury (SCI). Understanding the molecular mechanisms regulating microglial responses to injury may thus reveal novel therapeutic targets to promote CNS repair. Here, we investigated the role of microglial tumor necrosis factor receptor 2 (TNFR2), a transmembrane receptor previously associated with pro-survival and neuroprotective responses, in shaping the neuroinflammatory environment after CNS injury. By inducing experimental IS and SCI in *Cx3cr1*^CreER^:*Tnfrsf1b*^fl/fl^ mice, selectively lacking TNFR2 in microglia, and corresponding *Tnfrsf1b*^fl/fl^ littermate controls, we found that ablation of microglial TNFR2 significantly reduces lesion size and pro-inflammatory cytokine levels, and favors infiltration of leukocytes after injury. Interestingly, these effects were paralleled by opposite sex-specific modifications of microglial reactivity, which was found to be limited in female TNFR2-ablated mice compared to controls, whereas it was enhanced in males. In addition, we show that TNFR2 protein levels in the cerebrospinal fluid (CSF) of human subjects affected by IS and SCI, as well as healthy donors, significantly correlate with disease stage and severity, representing a valuable tool to monitor the inflammatory response after acute CNS injury. Hence, these results advance our understanding of the mechanisms regulating microglia reactivity after acute CNS injury, aiding the development of sex- and microglia-specific, personalized neuroregenerative strategies.

## 1. Introduction

Acute injury to the central nervous system (CNS), such as ischemic stroke (IS) and spinal cord injury (SCI), elicits rapid activation of microglia, the resident immune cells of the CNS, which promptly respond by proliferating and migrating to the lesioned area (Li et al., 2013; Raffaele and Fumagalli, 2022). Once recruited at lesion boundaries, microglia secrete cytokines and growth factors and increase their phagocytic clearance capacity to remove toxic debris, limit damage expansion, and promote tissue regeneration (Kawabori et al., 2015; Kroner et al., 2014). Single-cell transcriptomic studies have shown that acute CNS injury drives the formation of injury-responsive microglia populations, characterized by downregulation of homeostatic genes and upregulation of pathways related to phagocytosis, lipid metabolism, and protein secretion required for damaged tissue remodelling (Brennan et al., 2022; Matson et al., 2022; Zheng et al., 2022). However, excessive and prolonged inflammatory stimulation occurring at chronic stages of IS and SCI has been demonstrated to exhaust the pro-regenerative properties of microglial cells, which instead become dystrophic, acquire senescence-like traits, and contribute to damage progression and worsening of the functional outcome (Brennan et al., 2022; Raffaele et al., 2021). Indeed, at late injury stages, phagocytic microglia almost disappear and are replaced by cells displaying upregulation of pro-inflammatory and iron processing pathways, which are no longer able to sustain tissue repair (Brennan et al., 2022; Fu et al., 2023; Jin et al., 2023). On this basis, gaining a better understanding of the mechanisms that drive and maintain protective microglial responses to CNS injury may uncover novel therapeutic targets to promote tissue repair, by preventing the transition of microglia towards a dysfunctional and damaging state.

Production of tumor necrosis factor (TNF) by microglia is an essential feature of the early sterile immune response to acute CNS injury (Lambertsen et al., 2018; Lund et al., 2022a). TNF is synthesized as a transmembrane protein (tmTNF), then cleaved by the metalloprotease TNF-alpha converting enzyme (TACE/ADAM17) to release soluble TNF (solTNF) (Raffaele et al., 2020). Both tmTNF and solTNF exert their biological functions as homotrimers by activating two cognate receptors, TNF receptor 1 (TNFR1) and TNFR2, with TNFR1 responding to both solTNF and tmTNF, and TNFR2 preferentially responding to tmTNF, despite being able to bind also solTNF with high affinity (Grell et al., 1995; Grell et al., 1998). Both receptors are similar in their extracellular regions, while their intracellular domains exhibit striking structural differences which are reflected by partially overlapping and receptor type-specific signal transduction pathways (Atretkhany et al., 2020). Activation of TNFR1, but not TNFR2, is directly linked to cell death programs eliciting apoptosis and/or necroptosis (Probert, 2015). Conversely, TNFR2 lacks the intracellular death domain, and its stimulation triggers pro-survival and pro-regenerative responses (Probert, 2015). While TNFR1 is constitutively expressed on a broad spectrum of cell types, including CNS cells (Bette et al., 2003; Brambilla et al., 2011; Clausen et al., 2020; Hansen et al., 2021), TNFR2 is minimally expressed in homeostatic conditions, but upregulated in disease/stress states. It is largely expressed by immune cells and, within the CNS, it is mostly found in glial cells, with microglia being the highest expresser (Clausen et al., 2020; Hansen et al., 2021; Lambertsen et al., 2007; Lund et al., 2022a). Based on the distinct structural features and cellular expression profiles, several experimental studies have highlighted the opposite role of TNFRs in neurological diseases, with TNFR1 mediating dominantly the pro-inflammatory activities of TNF, and TNFR2 being primarily involved in neuroprotective and neuroreparative processes through tmTNF binding (Brambilla et al., 2011; Probert, 2015).

In this respect, previous studies from our group demonstrated that microglial TNFR2 signaling mediates pro-regenerative responses in CNS demyelinating disease by regulating microglial phagocytosis and sensing capabilities (Gao et al., 2017). Consistent with these findings, inflammatory stimulation of microglia has been reported to trigger the production of anti-inflammatory mediators in a TNFR2-dependent manner (Veroni et al., 2010). Nevertheless, whether similar TNFR2-dependent protective mechanisms are at play following acute CNS injury as well remained to be investigated. Here, to fill this gap, we induced experimental IS and SCI in *Cx3cr1*^CreER^:*Tnfrsf1b*^fl/fl^ mice with conditional ablation of microglial TNFR2 and corresponding *Tnfrsf1b*^fl/fl^ littermate controls (Gao et al., 2017). By this mean, we demonstrate that ablation of microglial TNFR2 affects microglia recruitment at the lesion site and their phagocytic phenotype in an opposite sex-specific fashion, leading to significant changes in lesion size, pro-inflammatory cytokine levels, and infiltration of leukocytes after injury. In addition, we show that quantification of TNFR2 protein levels in the cerebrospinal fluid (CSF) of human subjects affected by IS and SCI, as well as healthy donors, allows to discriminate participants based on disease stage and severity, possibly reflecting the extent of microglial activation within the injury site.

## 2. Materials and methods

### 2.1. Animals and experimental procedures

#### 2.1.1. Mice

Adult (12–17 weeks) *Cx3cr1*^CreER^:*Tnfrsf1b*^fl/fl^ conditional knockout mice, in which consistent TNFR2 ablation is achieved specifically in microglia (Gao et al., 2017), and control *Tnfrsf1b*^fl/fl^ littermates with normal TNFR2 expression have been compared in this study.

For the experimental stroke study, breeding couples were transferred from The Miami Project to Cure Paralysis, University of Miami Miller School of Medicine, Miami, FL to the Biomedical Laboratory, Institute of Molecular Medicine, University of Southern Denmark, where they were kept as a colony. Mice were cared for in accordance with the protocols and guidelines approved by the Danish Veterinary and Food Administration (J. No. 2013–15-2934–00924 and J. No. 2019–15-0201–01620).

For the experimental spinal cord injury (SCI) study, breeding couples were transferred from the University of Southern Denmark to South China Agricultural University. Mice were cared for in accordance with the protocols and guidelines approved by the South China Agricultural University (#SYXK2019-0136).

Animals were housed under conditions with diurnal lighting, controlled temperature and humidity, and with food and water available ad libitum. All experiments are reported in agreement with the ARRIVE 2.0 guidelines and all efforts were made to minimize pain and distress.

Genotyping was performed on DNA extracts from ear biopsies as previously described (Gao et al., 2017). To induce recombination, 8–13 weeks old mice received 5 consecutive intraperitoneal injections of 1 mg/day tamoxifen (MP Biomedicals LLC, Roskilde, Denmark) dissolved in sunflower oil (Sigma-Aldrich, Søborg, Denmark), followed by a 28-days waiting period to allow drug wash-out and full replenishment of TNFR2-expressing circulating leukocytes.

#### 2.1.2. Cell cultures

Primary microglia and bone marrow-derived macrophages (BMDMs) were isolated from naïve *Cx3cr1*^CreER^:*Tnfrsf1b*^fl/fl^ mice and *Tnfrsf1b*^fl/fl^ controls (n = 3/group) 28 days after tamoxifen treatment.

Primary microglia cultures were obtained from dissected brain tissue using the Adult Brain Dissociation Kit (Miltenyi Biotec, Bergisch Gladbach, Germany), anti-CD11b microbeads (Miltenyi Biotec) and MACS LS columns (Miltenyi Biotec) according to the manufacturer’s protocol. Microglia were kept in culture for 6 days as previously described (Yli-Karjanmaa et al., 2019b).

Bone marrow precursor cells (BMPCs) were isolated from the femoral and tibial bone marrow. Red blood cells were removed using red blood cell lysis buffer (Thermo Fisher Scientific, Slangerup, Denmark) according to the manufacturer’s protocol. BMPCs were cultivated at 4.44 million cells/T-25 flask in pre-warmed 88 % Dulbecco’s modified eagle medium (DMEM) containing 10 % fetal bovine serum (FBS), 1 % penicillin/streptomycin, and 1 % glutamax (all from Thermo Fischer Scientific) along with 20 ng/ml macrophage colony-stimulating factor (M–CSF; Sigma-Aldrich) to stimulate differentiation into BMDMs for 7 days total.

Cell cultures were maintained in a humidified incubator (5 % CO_2_, 21 % O_2_) at 37 °C and stimulated with 100 ng/mL lipopolysaccharide (LPS from Escherichia coli 0111:B4; Thermo Fisher Scientific) for 24 h before harvesting for electrochemiluminescence analysis as described below.

#### 2.1.3. Permanent middle cerebral artery occlusion (pMCAO)

Permanent left middle cerebral artery occlusion (pMCAO) was performed as previously described (Gregersen et al., 2000; Lambertsen et al., 2009). Briefly, female and male mice were anesthetized with a 1:1:2 ratio of Hypnorm (fentanyl citrate 0.315 mg/ml and fluanisone 10 mg/ml; VetaPharma Ltd, Leeds, UK), Midazolam (5 mg/ml midazolam hydrochloride; Hameln Pharma, Hameln, Germany), and distilled water. The skin between the orbit and the ear was vertically incised with a scalpel, the temporal muscle was dissected, and a 1 mm hole was created in the skull using a drill to expose the MCA. The left MCA was then permanently occluded by means of microbipolar coagulation. After pMCAO, the mice were sutured, injected with saline to prevent dehydration, and kept in a 28 °C heating cabinet for 24 h to allow recovery from surgical procedures. Buprenorphine hydrochloride (0.001 mg/20 g Temgesic) was administered 3 times at 8 h intervals for post-surgical analgesia, starting at the time of surgery.

#### 2.1.4. Contusive spinal cord injury (SCI)

SCI was induced with the mouse Infinite Horizon-0400 SCI Contusion Device (Precision Systems and Instrumentation, LLC, Brimstone, LN, USA) as previously described (Lund et al., 2022b; Novrup et al., 2014). Briefly, female mice received a laminectomy at thoracic vertebrae 8, and a 75kD contusion was given. Following SCI, mice were sutured and injected with saline to prevent dehydration and buprenorphine hydrochloride (0.001 mg/20 g Temgesic) and antibiotic gentamicin (40 mg/kg) for post-surgical analgesia once per day for the first 7 days. Mice were housed separately in a recovery room, where their post-surgical health status was monitored during a 24–8 h recovery period. Manual bladder expression was performed twice a day for the first 7 days and then changed to once daily for the rest of the study; male mice were not included in SCI experiments due to high risk of bladder infection and rupture related to this procedure. Body weight and locomotor recovery were monitored weekly. Locomotor recovery was assessed using the Basso Mouse Scale (BMS; (Basso et al., 2006)).

### 2.2. Behavioral tests

All behavioral tests were performed at University of Southern Denmark, with the observer blinded to the genotype. Mice were given at least one day off between tests, and the time occurring between trials for each test is detailed below.

#### 2.2.1. Open field

The open field test was performed to assess anxiety-related behavior and locomotor activity in naïve *Cx3cr1*^CreER^:*Tnfrsf1b*^fl/fl^ mice and *Tnfrsf1b*^fl/fl^ littermates (n = 11/group) as previously described (Lambertsen et al., 2012b). Mouse behavior in the open field arena was recorded over a 10 min period using the SMART 3.0 Video Tracking System (Panlab Harvard Apparatus, Barcelona, Spain) connected to a high-resolution color video camera (SSC-DC378P, Biosite, Stockholm, Sweden). The total distance travelled, and the percentage time spent in wall, intermediate, and center zone, were automatically recorded. Center and wall rearing was manually recorded and expressed as total number of events as a measure of stereotypical mouse behavior.

#### 2.2.2. Elevated plus maze

The elevated plus maze was used to assess anxiety-related behavior in naïve *Cx3cr1*^CreER^:*Tnfrsf1b*^fl/fl^ mice and *Tnfrsf1b*^fl/fl^ littermates (n = 11/group) as previously described (Ellman et al., 2017). During a 5 min test, the total distance travelled, and the time spent in the closed and open arms were recorded using the SMART video tracking software (Panlab). An open/closed arm ratio was calculated based on the times spent in each arm as a measure of anxiety-related behavior.

#### 2.2.3. Grip strength

The grip strength measuring device (BIO-GT3, Bioseb) was used to measure total (both right and left) and side-specific (right or left only) forepaw strength in *Cx3cr1*^CreER^:*Tnfrsf1b*^fl/fl^ mice and *Tnfrsf1b*^fl/fl^ littermates (n = 14–18/group) at baseline and day 1 post-pMCAO as previously described (Clausen et al., 2016). Results are presented as percentage of baseline values to account for individual differences between mice.

#### 2.2.4. Hargreaves test

The Hargreaves nociception assay was performed to evaluate thermal hyperalgesia in *Cx3cr1*^CreER^:*Tnfrsf1b*^fl/fl^ mice and *Tnfrsf1b*^fl/fl^ littermates (n = 10–14/group) at baseline, day 3, and 5 post-pMCAO as previously described (Yli-Karjanmaa et al., 2019a). Briefly, the plantar test (37370, Ugo Basile, Comerio VA, Italy) was used to measure the right and left hind paw withdrawal latency during five trials separated by 15 min recovery. Results are presented as percentage of baseline.

### 2.3. Tissue processing

At the selected time points, mice undergoing pMCAO were deeply anaesthetized with pentobarbital (200 mg/ml) containing lidocaine (20 mg/ml) and euthanized by cervical dislocation, and the brains were dissected and snap frozen using carbon dioxide snow. Parallel series of 30 μm-thick coronal sections were cut using a cryostat and placed on gelatin-coated slides for infarct volume estimation or collected in tubes for electrochemiluminescence, qPCR, and western blotting analyses.

### 2.4. Lesion volume analysis

#### 2.4.1. Ischemic infarct volume

For the estimation of ischemic infarct volumes, one series of brain sections from *Cx3cr1*^CreER^:*Tnfrsf1b*^fl/fl^ mice and *Tnfrsf1b*^fl/fl^ littermates, collected at day 1 and 5 post-pMCAO (n = 8–10/group), were stained with Toluidine Blue (Clausen et al., 2006). Direct infarct volumes were estimated using the computer-assisted stereological test system CAST 2000 (Olympus, Ballerup, Denmark) and applying Cavalieri’s principle, as previously described (Gregersen et al., 2000). Indirect infarct volumes were estimated by subtracting the volume of the healthy ipsilateral cortex from the contralateral cortex (Madsen et al., 2016a). The rostrocaudal distribution of the infarct was calculated based on the anterior commissure, as previously described (Lambertsen et al., 2004).

#### 2.4.2. SCI lesion area

For SCI lesion volume analysis, spinal cord segments obtained from *Cx3cr1*^CreER^:*Tnfrsf1b*^fl/fl^ mice and *Tnfrsf1b*^fl/fl^ littermates at day 28 post-SCI (n = 4–6/group) were longitudinally sectioned into 10-μm thick serial sections. After staining for glial fibrillary acidic protein (GFAP) as described below, GFAP negative areas from three serial sections at 100 μm intervals with lesion epicenter in the middle were outlined and quantified with ImageJ software. Average areas from those three sections were used to estimate lesion areas as previously described (Brambilla et al., 2005).

### 2.5. Electrochemiluminescence analysis

Electrochemiluminescence assays were used to quantify the protein levels of TNF receptors, cytokines and chemokines in human CSF samples, mouse brain tissue lysates, and cell lysates from primary cultured murine BMDMs and microglia, as previously described (Byg et al., 2022; Madsen et al., 2016b; Yli-Karjanmaa et al., 2019a). Briefly, tissue samples were processed in Complete Mesoscale Lysis Buffer (Mesoscale Discovery) and protein concentrations were measured by Micro BCA^™^ Protein Assay Kit (Thermo Fisher Scientific), both according to the manufacturer’s protocol. TNFR2 levels were quantified using the Human or Mouse TNFRII Ultra-Sensitive Kit (Mesoscale Discovery). TNFR1 levels were measured using the Mouse TNFR1 Ultra-Sensitive Kit (Mesoscale Discovery). Cytokine (TNF, interferon (IFN)γ, interleukin (IL)-1β, IL-2, IL-4, IL-5, IL-6, IL-10, IL-12p70) and chemokine (CXCL1, CCL-5) levels were assessed using the custom-made MSD Mouse Pro-inflammatory V-PLEX and Ultra PLEX kits (Mesoscale Discovery). All analyses were performed in duplicate on a SECTOR Imager 6000 Plate Reader (Mesoscale Discovery). Samples with coefficient of variation (CV) values > 25 % in individual analyses were excluded. The lower limit of detection (LLOD) was a calculated concentration-based on a signal 2.5 standard deviations (SD) above the blank (zero) calibrator. For protein levels below LLOD, a value of 0.5 LLOD was used for statistical analysis (Madsen et al., 2016a; Yli-Karjanmaa et al., 2019a).

### 2.6. RNA isolation and real-time PCR

The analysis of relative gene expression *in vivo* was performed on bulk brain tissue obtained from *Cx3cr1*^CreER^:*Tnfrsf1b*^fl/fl^ mice and *Tnfrsf1b*^fl/fl^ littermates prior to ischemia induction (naïve, n = 5) and at day 1 (n = 10/group) and day 5 (n = 10/group) post-pMCAO. The analysis of microglial *Tnfrsf1b* gene expression was performed on cells isolated from the ipsilateral cortex of *Cx3cr1*^CreER^:*Tnfrsf1b*^fl/fl^ mice and *Tnfrsf1b*^fl/fl^ littermates at day 5 post-pMCAO (n = 4–6/group) using the Adult Brain Dissociation Kit (Miltenyi Biotec), anti-CD11b microbeads (Miltenyi Biotec) and MACS LS columns (Miltenyi Biotec) according to the manufacturer’s protocol.

Total RNA was extracted from bulk brain tissue and MACS-sorted CD11b^+^ cells using TRIZOL^®^ reagent (Thermo Fischer Scientific) and chloroform-isopropanol (Sigma-Aldrich) phase separation. Synthesis of cDNA was performed using the High-Capacity cDNA Reverse Transcription kit (Thermo Fisher Scientific) following manufacturer’s instructions.

RT-qPCR was performed using the following primers (TAG, Copenhagen, Denmark): *Cd68* (Fw: GGTGGAAGAAAGGCTTGGGG; Rv: GAGACAGGTGGGGATGGGTA), *Hprt1* (Fw: TCCTCAGACCGCTTTTTGCC; Rv: TCATCATCGCTAATCACGACGC), *Tnfrsf1b* (Fw: GCCCAGCCAAACTCCAAGCATC; Rv: TCCTAACATCAGCAGACCCAGTG), and *Rpl13a* (Fw: ACAGCCACTCTGGAGGAGAA; Rv: GAGTCCGTTGGTCTTGAGGA). SYBR Green Gene Expression Assay was utilized to quantify gene expression using a CFX96 real-time PCR system. The relative transcript levels were calculated using the Pfaffl method (Pfaffl, 2001) and normalized to the housekeeping genes *Hprt1* for *Cd68* and *Rpl13a* for *Tnfrsf1b*. Data are presented as mean of log_2_(fold change) ± SEM.

### 2.7. Western blotting

Western blotting analysis was performed as previously described (Orti-Casan et al., 2022) on bulk brain tissue obtained from *Cx3cr1*^CreER^:*Tnfrsf1b*^fl/fl^ mice and *Tnfrsf1b*^fl/fl^ littermates prior to ischemia induction (naïve, n = 5/group) and 3 h post-pMCAO (n = 5/group). Samples were lysed by addition of 250 μL lysis buffer (50 mM Tris·HCl, pH 7.4; 1 % Nonidet P-40; 0.25 % sodium deoxycholate; 150 mM NaCl; 1 mM ethylenediaminetetraacetic acid; complete mini protease-inhibitor mixture tablet). Tissue lysates were centrifuged at 4 °C (30 min at 9,600g), and the protein concentration of supernatants was measured by the Bradford assay and adjusted to 2 μg/μL. A total of 20 μg protein was denatured in Laemmli buffer for 10 min at 70 °C and separated by 4–12 % sodium dodecyl sulfate–polyacrylamide gel electrophoresis (160 V, 40 min). Proteins were then transferred to polyvinylidene difluoride membranes and blocked with 5 % skim-milk powder solution in Tris-buffered saline (TBS) containing 0.1 % Tween 20 for 1 h. Subsequently, membranes were incubated overnight at 4 °C with the following primary antibodies: anti–phospho-Akt Ser-473 (1:500; #9271, Cell Signaling) or anti-Akt (1:2,000; #9272, Cell Signaling). Then, membranes were washed with TBS containing 0.1 % Tween 20 and incubated with horseradish peroxidase–conjugated secondary antibody goat anti-rabbit (1:10,000; #7074, Cell Signaling) in TBS containing 0.1 % Tween 20 for 2 h at room temperature. Immunoreactivity was detected using enhanced chemiluminescence (Pierce Biotechnology) with a ChemiDoc XRS system (Bio-Rad Laboratories). Glyceraldehyde-3-phosphate dehydrogenase (GAPDH; 1:3,000; #MA1-16757, Invitrogen) expression was analyzed as loading control.

### 2.8. Flow cytometry

Flow cytometry analysis was performed on the ipsilateral cerebral cortex of *Cx3cr1*^CreER^:*Tnfrsf1b*^fl/fl^ mice and *Tnfrsf1b*^fl/fl^ littermates at day 5 post-pMCAO (n = 5–7/group) and on the spinal cord collected at day 7 post-SCI (n = 3–4/group). Mice were deeply anaesthetized with pentobarbital (200 mg/ml) containing lidocaine (20 mg/ml), transcardially perfused with 20 ml of ice-cold 0.01 M phosphate-buffered saline (PBS), and the ipsilateral cortex or thoracic spinal cord were dissected and placed in cold RPMI-1640 (Gibco, Roskilde, Denmark). Samples were homogenized through a 70 μm cell strainer (AH Diagnostics, Aarhus, Denmark) and myelin and cell debris were removed using the Debris Removal Solution (Miltenyi Biotec) according to manufacturer’s protocol.

For IS studies, analysis was carried out using the following panel: PerCP-Cy5.5 rat anti-mouse CD45 (clone 30-F11, BD Biosciences, Eysins, Switzerland), FITC rat anti-CD11b (clone M1/70, BD Biosciences), BV421 rat anti-mouse LY-6G (clone 1A8, BD Biosciences), and their corresponding isotype controls. Briefly, after myelin removal, the cell suspensions were stained for live/dead cells using Fixable Viability Dye eFlouro 506 (eBioscience, San Diego, CA, USA), blocked using purified rat anti-mouse CD16/CD32 (Mouse BD Fc block; BD Biosciences) and Syrian hamster gamma globulin (Jackson Immuno Research Europe Ltd, Cambridge, UK) for 30 min at 4 °C. Cells where then washed and fixed with 2 % paraformaldehyde (PFA) for 15 min. After fixation, the samples were blocked and stained for CD45/CD11b/Ly-6G. Trucount^™^ beads (BD Biosciences, Eysins, Switzerland) were added to each sample for the quantification of absolute cell numbers. Stained cells were run on a FACSAria^™^ III flow cytometer (BD Biosciences, Eysins, Switzerland), and approximately 50,000–100,000 events were acquired per sample using forward scatter (FSC) and side scatter (SSC). The analysis was performed using the Flowlogic software, determining positive staining based on the respective isotype and fluorescent minus one (FMO) control, and automatically determining the absolute number of positive events, percentage of parental cell population, and geometric mean fluorescence intensity (MFI) (Clausen et al., 2016).

For SCI studies, analysis was carried out using the following panel: PE-Cy7 rat anti-mouse CD45 (clone 30-F11, eBioscience), eFluor450 rat anti-mouse CD11b (clone M1/70, eBioscience), PE rat anti-mouse CD4 (clone RM4-5, eBioscience), APC rat anti-mouse CD8 (clone SK1, eBioscience), APC-eFluor780 rat anti-mouse B220 (clone RA3-6B2, eBioscience), APC-Cy7 rat anti-mouse CD19 (clone 1D3, BD Biosciences), FITC mouse anti-mouse NK1.1 (clone PK136, eBioscience), PerCP-Cy5.5 rat anti-mouse Ly6G (clone RB6-8C5, eBioscience). After myelin removal, cells were resuspended in 100 μL FACS buffer, blocked with anti-CD16/32 (FcR block, eBioscience) on ice for 10 min, immunostained for 30 min at 4 °C, and fixed with 1 % PFA. Cells were analyzed with an LSRII flow cytometer equipped with FACS-Diva 6.0 software (BD Biosciences) as previously described (Brambilla et al., 2014). The number of spinal cord leukocytes was determined with 123count eBeads (eBioscience).

### 2.9. Immunohistochemistry

Immunohistochemistry (IHC) analysis has been performed on *Cx3cr1*^CreER^:*Tnfrsf1b*^fl/fl^ mice and *Tnfrsf1b*^fl/fl^ littermates euthanized at day 1 (n = 3/group) and 5 post-pMCAO (n = 5–6/group) or at day 28 post-SCI (n = 4–6/group). Mice were deeply anesthetized with pentobarbital (200 mg/ml) containing lidocaine (20 mg/ml) and transcardially perfused with 0.01 M PBS followed by 4 % PFA in 0.01 M PBS. Brains were dissected, post-fixed for 1 h in 4 % PFA and cryoprotected in a solution containing 30 % sucrose in 0.01 M PBS at 4 °C until precipitation. Coronal sections of 20 μm thickness were collected using a cryostat and incubated overnight at 4 °C in 0.01 M PBS with 1 % goat serum (Dako, Glostrup, Denmark) and 0.3 % Triton X-100 with the following primary antibodies: rat anti-CD68 (1:400; BioRad Laboratories, Segrate, Italy); chicken anti-GFAP (1:1000, Abcam, USA); rabbit anti-Iba1 (1:500; Wako, Japan); goat anti-TNFR2 (1:50, R&D Systems, Abingdon, UK). The sections were then exposed for 2 h at room temperature to the corresponding secondary antibodies conjugated with Alexa Fluor 488 and 594 (1:600; Life Technologies, Monza, Italy). Nuclei were labeled with 4′,6-diamidino-2-phenylindole (DAPI). For the quantitative analysis, the *peri*-infarct region (0–500 μm from the border of the ischemic lesion) and the *peri*-lesion area (0–500 μm from the border of the SCI lesion) were considered. Images were acquired using a confocal microscope (merge of 6-μm z-stack at 2-μm intervals; A1R, Nikon, Tokyo, Japan) and a fluorescence microscope (BX53 fluorescence microscope fitted with DP73 camera, Olympus, Tokyo, Japan).

Image analysis was performed by an investigator blinded to genotypes, using the software Fiji/ImageJ, on three slices per mouse taken from – 1.00 to 0.00 mm from bregma, as previously described (Raffaele et al., 2021). The density of Iba1^+^ and Iba1^+^CD68^+^ cells was determined by dividing manually counted positive cell numbers on the area examined. The percentage of CD68^+^ cells was calculated by dividing the number of Iba1^+^CD68^+^ cells on the total Iba1^+^ cells. The area fraction covered by Iba1 and CD68 staining, and the average size of Iba1^+^ cells, were automatically determined using the particle analysis tool of the Fiji-ImageJ software, as previously described (Lund et al., 2023; Magni et al., 2020), and are expressed as fold over values obtained in control mice set to 100.

### 2.10. Human CSF samples collection

Human cerebrospinal fluid (CSF) samples from individuals with stroke-related symptoms were obtained at the University Medical Center Hamburg-Eppendorf, Germany (Supplementary Table 1). The samples were divided into “minor stroke” and “major stroke” according to the size and configuration of the ischemia on the cerebral imaging. All procedures were approved by the local ethics committee at the University Medical Center Hamburg-Eppendorf (PV5340), and informed consent was obtained from all patients or their legal representatives. Lumbar puncture was performed within the first days after stroke as part of the regular diagnostic procedures. After extraction, CSF was centrifuged and the cell-free supernatants were collected, aliquoted, and frozen (<30 min after taking) at −80 °C until use.

Human CSF samples from healthy controls were collected after at least 2 h of fasting at Odense University Hospital, Denmark (Supplementary Table 1). Ethical approval was granted by the local research ethics committee (The Regional Committees on Health Research Ethics for Southern Denmark: J. no. S-20160173) and informed written consent was obtained from all participants. Pain-free individuals with no neurological, infectious, malignant, psychiatric, or immunological diseases or substance abuse were included (Blichfeldt-Eckhardt et al., 2023). After extraction, CSF samples were centrifuged, aliquoted, frozen (<30 min after taking), and stored at −80 °C until use.

Human CSF samples from individuals with SCI were collected and stored at the Third Affiliated Hospital of Sun Yat-sen University in China (Supplementary Table 2). All individuals received a diagnosis of SCI based on clinical symptoms (ISNCSCI and ASIA score), electrophysiology, x-ray, and MRI analysis. Cases were divided into acute (2 weeks-2 months after injury), early chronic (2–12 months after injury), and late chronic stages (>24 months after injury) (Gao et al., 2023). SCI cases with complete or incomplete traumatic injury at cervical and thoracic levels were included in this study. One case had a lumbar SCI. Individuals with concomitant neurological disorders or diabetes were excluded. CSF from healthy individuals was used as control, and samples were collected after overnight fasting, centrifuged, and frozen (<30 min after taking) at −80 °C until use. The study protocols ([2018]-02, [2018]-03, [2018]-04) were in accordance with guidelines for clinical studies approved by the Third Affiliated Hospital of Sun Yat-sen University review board.

### 2.11. Human tissue staining

Human post-mortem tissue encompassing right parietal lobe brain tissue derived from a 59-year old male with a > 7 days old infarct was stained using protocols previously described in detail (Clausen et al., 2020) using rabbit anti-TNFR2 (1:50, Sigma-Aldrich) and mouse anti-Iba1 (1:1,000 Sigma-Aldrich) antibodies. The use of human post-mortem brain tissue was approved by the Regional Committees on Health Research Ethics for Southern Denmark (Journal number S-20220018) and the project was reported to the Danish Data Protection Agency.

### 2.12. Statistical analyses

For each experimental procedure, the proper sample size was calculated by mean of the G*Power 3.1 software, estimating effect size and standard deviation based on previously published data and fixing the alpha value (type 1 error) at the level of 5 % (p = 0.05) and the power at 80 %.

Data are expressed as mean ± standard error of mean (SEM). Statistical analysis was performed using the Prism 7 software (GraphPad, San Diego, CA, USA). Gaussian distribution of the values in each experimental group has been assessed using Shapiro-Wilk (for n < 8) or D’Agostino-Pearson (for n > 8) normality tests. For all comparisons between two groups with a normal distribution, unpaired Student’s *t*-test was performed, while for groups without normal distribution nonparametric Mann-Whitney test was used. For multiple comparison testing, depending on the experimental design, one-way or two-way analysis of variance (ANOVA) accompanied by Tukey’s or other appropriate post-hoc test was used for groups with normal distribution, whereas nonparametric Kruskal-Wallis test followed by Dunn’s post-hoc analysis was performed when normal distribution of the values could not be assumed. For correlation analysis, two-tailed Pearson test was used. Differences were considered significant for p-value < 0.05.

### 2.13. Data availability

Further information and data that support the findings of this study are available from the corresponding author upon reasonable request.

## 3. Results

### 3.1. Ablation of TNFR2 from microglia limits lesion size after ischemic stroke and spinal cord injury

To evaluate the contribution of microglial TNFR2 signaling in the pathophysiology of IS and SCI, we used *Cx3cr1*^CreER^:*Tnfrsf1b*^fl/fl^ conditional knockout mice generated and characterized in our laboratories, that allow for selective ablation of TNFR2 in microglial cells (Gao et al., 2017). Here, we further verified conditional ablation of microglial *Tnfrsf1b* by demonstrating absence of TNFR2 protein in LPS-stimulated primary microglia derived from *Cx3cr1*^CreER^:*Tnfrsf1b*^fl/fl^ mice, which was instead present in LPS-stimulated microglia derived from *Tnfrsf1b*^fl/fl^ littermates (Supplementary Fig. 1A). Confirming the selectivity of microglial ablation in our model, TNFR2 expression was unaffected in LPS-stimulated bone marrow derived macrophages (BMDMs) isolated from *Cx3cr1*^CreER^:*Tnfrsf1b*^fl/fl^ or *Tnfrsf1b*^fl/fl^ mice (Supplementary Fig. 1B).

**Fig. 1.**
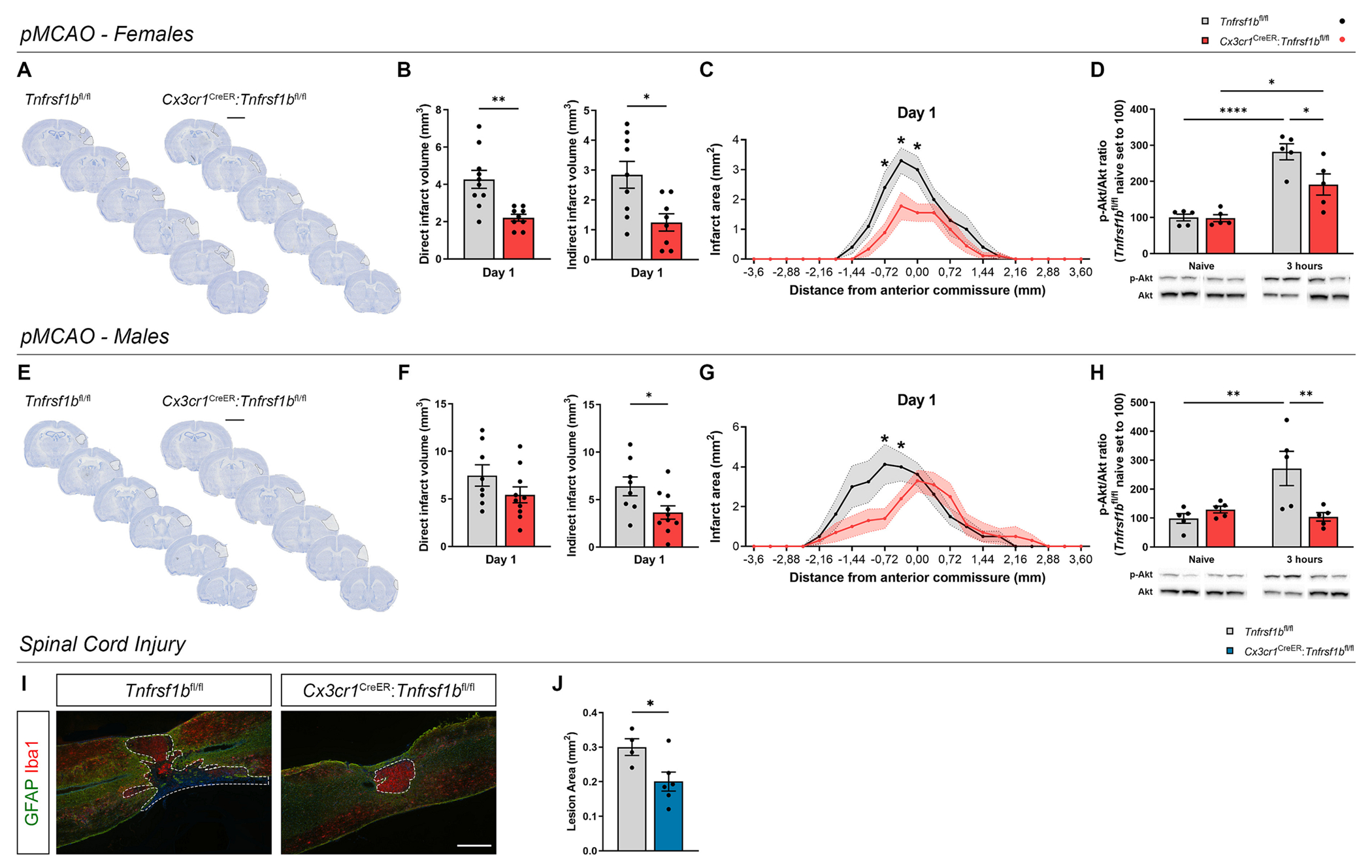
Conditional ablation of microglial TNFR2 affects lesion size in experimental models of ischemic stroke and spinal cord injury. (A) Representative images of toluidine blue-stained brain sections from female *Cx3cr1*^CreER^:*Tnfrsf1b*^fl/fl^ mice and *Tnfrsf1b*^fl/fl^ littermates at day 1 post-permanent middle cerebral artery occlusion (pMCAO). Scalebar: 5 mm. (B) Quantification of the direct and indirect infarct volumes in female *Cx3cr1*^CreER^:*Tnfrsf1b*^fl/fl^ mice and *Tnfrsf1b*^fl/fl^ littermates at day 1 post-pMCAO (direct: n = 9–10; indirect: n = 8–9). * p < 0.05, ** p < 0.01; Student’s *t*-test. (C) Rostrocaudal distribution of the ischemic infarct in female *Cx3cr1*^CreER^:*Tnfrsf1b*^fl/fl^ mice and *Tnfrsf1b*^fl/fl^ littermates at day 1 post-pMCAO (n = 9–10). * p < 0.05; Multiple *t*-test. (D) Quantification of p-Akt/Akt ratio in the brain of female *Cx3cr1*^CreER^:*Tnfrsf1b*^fl/fl^ mice and *Tnfrsf1b*^fl/fl^ littermates in naïve conditions and at 3 h post-pMCAO (n = 5/group). * p < 0.05, **** p < 0.0001; Two-way ANOVA (Interaction*, Time****, Genotype*) followed by Sidak’s multiple comparisons test. (E) Representative images of toluidine blue-stained brain sections from male *Cx3cr1*^CreER^:*Tnfrsf1b*^fl/fl^ mice and *Tnfrsf1b*^fl/fl^ littermates at day 1 post-pMCAO. Dashed lines delineate the ischemic infarct. Scalebar: 5 mm. (F) Quantification of the direct and indirect infarct volumes in male *Cx3cr1*^CreER^:*Tnfrs1b*^fl/fl^ mice and *Tnfrsf1b*^fl/fl^ littermates at day 1 post-pMCAO (direct: n = 8–10; indirect: n = 8–10). * p < 0.05; Student’s *t*-test. (G) Rostrocaudal distribution of the ischemic infarct in male *Cx3cr1*^CreER^:*Tnfrsf1b*^fl/fl^ mice and *Tnfrsf1b*^fl/fl^ littermates at day 1 post-pMCAO (n = 9–10). * p < 0.05; Multiple *t*-test. (H) Quantification of p-Akt/Akt ratio in the brain of male *Cx3cr1*^CreER^:*Tnfrsf1b*^fl/fl^ mice and *Tnfrsf1b*^fl/fl^ littermates in naïve conditions and at 3 h post-pMCAO (n = 5/group). ** p < 0.01; Two-way ANOVA (Interaction*, Time****, Genotype*) followed by Sidak’s multiple comparisons test. (I) Representative images of spinal cord lesions stained with the microglia/macrophage marker Iba1 and the astrocyte marker GFAP in female *Cx3cr1*^CreER^:*Tnfrsf1b*^fl/fl^ mice and *Tnfrsf1b*^fl/fl^ littermates at day 28 after spinal cord injury (SCI). Dashed lines delineate SCI lesions. Scalebar: 200 μm. (J) Quantification of lesion area in female *Cx3cr1*^CreER^:*Tnfrsf1b*^fl/fl^ mice and *Tnfrsf1b*^fl/fl^ littermates at day 28 after SCI (n = 4–6). * p < 0.05; Student’s *t*-test. (For interpretation of the references to color in this figure legend, the reader is referred to the web version of this article.)

To test whether ablation of microglial TNFR2 affected ischemic lesion formation, *Tnfrsf1b*^fl/fl^ and *Cx3cr1*^CreER^:*Tnfrsf1b*^fl/fl^ mice were subjected to permanent middle cerebral artery occlusion (pMCAO), and direct and indirect infarct volumes were quantified on day 1 and 5 after the surgery by toluidine blue staining. In female mice, both direct and indirect infarct volumes were significantly reduced in microglial TNFR2-ablated mice compared to controls at day 1 post-pMCAO (Fig. 1A–B). This was confirmed by the rostrocaudal distribution of the infarcts, showing a significantly smaller rostral lesion size following ablation of microglial TNFR2 at day 1 post-pMCAO (Fig. 1C). No significant differences were instead observed between genotypes in direct and indirect infarct volumes, nor in the rostrocaudal distribution of the infarcts, a day 5 post-pMCAO (Supplementary Fig. 2A–C). Because PI3K-PKB/Akt is a key pathway activated downstream of TNFR2 and contributing to TNFR2-dependent neuroprotective and immunoregulatory functions (Dong et al., 2016; Fu et al., 2021), we assessed PI3K-PKB/Akt signaling activation by measuring Akt and phosphorylated Akt (p-Akt) in brain lysates from female *Cx3cr1*^CreER^:*Tnfrsf1b*^fl/fl^ mice and *Tnfrsf1b*^fl/fl^ littermates in naïve conditions and 3 h post-pMCAO by western blotting. Akt signaling activation, measured as pAkt/Akt ratio, was significantly increased in both microglial TNFR2-ablated mice and *Tnfrsf1b*^fl/fl^ littermates 3 h post-pMCAO compared to naïve conditions (Fig. 1D and Supplementary Fig. 3A). However, activation was significantly lower in *Cx3cr1*^CTeER^:*Tnfrsf1b*^fl/fl^ compared to *Tnfrsf1b*^fl/fl^ littermates (Fig. 1D), indicating a suppression of TNFR2-dependent responses in TNFR2 ablated mice, as anticipated.

Noteworthy, at day 1 post-pMCAO, we found a significant reduction only of indirect infarct volume in male *Cx3cr1*^CreER^:*Tnfrsf1b*^fl/fl^ mice compared to *Tnfrsf1b*^fl/fl^ littermates, which was mainly localized in the rostral part of the lesion, while no marked differences were observed in the direct infarct volume (Fig. 1E–G). Moreover, at day 5 post-pMCAO, no significant differences were found between genotypes in both direct and indirect infarct volumes, while rostrocaudal analysis showed that the infarcts were slightly bigger in the rostral part of the brain in *Cx3cr1*^CTeER^:*Tnfrsf1b*^fl/fl^ mice compared to controls (Supplementary Fig. 2D–F). In male mice, we observed a significant increase of pAkt/Akt ratio in *Tnfrsf1b*^fl/fl^ littermates 3 h post-pMCAO compared to naïve, which was totally suppressed in *Cx3cr1*^CTeER^:*Tnfrsf1b*^fl/fl^ mice, confirming the inhibition of TNFR2-dependent intracellular cascade (Fig. 1H and Supplementary Fig. 3B).

Since regional diversity in microglial functions has been described between brain and spinal cord (Pavic et al., 2021), the contribution of microglial TNFR2 to lesion evolution may be divergent in distinct CNS regions and pathologies. To address this question, *Tnfrsf1b*^fl/fl^ and *Cx3cr1*^CreER^:*Tnfrsf1b*^fl/fl^ female mice were subjected to a different type of traumatic CNS injury, mild experimental SCI (Novrup et al., 2014), and euthanized at day 28 to measure lesion area. Notably, microglial TNFR2-ablated female mice displayed smaller spinal cord lesions compared to controls (Fig. 1I, J). Altogether, these findings demonstrated that, in female mice, conditional ablation of TNFR2 in microglia similarly decreased lesion size in the brain and spinal cord after pMCAO and SCI, respectively.

### 3.2. Microglial TNFR2 ablation has no impact on functional deficits after ischemic stroke and spinal cord injury

Since both IS and SCI lead to sensory-motor deficits, we first assessed whether microglial TNFR2 ablation itself influenced basal locomotor function and exploratory activity prior to injury (Supplementary Fig. 4). Motor coordination was previously evaluated by rotarod test and resulted to be normal in naïve *Cx3cr1*^CreER^:*Tnfrsf1b*^fl/fl^ mice compared to *Tnfrsf1b*^fl/fl^ littermates (Gao et al., 2017). Locomotor activity, exploratory behavior, and anxiety traits were assessed by open field and elevated plus maze tests. In the open field test, total distance travelled and rearing activity were increased in female *Cx3cr1*^CTeER^:*Tnfrsf1b*^fl/fl^ mice compared to controls, while the time spent in the wall zone of the field was reduced (Supplementary Fig. 4A). Conversely, in male *Cx3cr1*^CreER^:*Tnfrsf1b*^fl/fl^ mice only a significant increase of center rearing was found (Supplementary Fig. 4B). In the elevated plus maze, a significant increase of the total distance travelled was observed in female but not male *Cx3cr1*^CreER^:*Tnfrsf1b*^fl/fl^ mice, while in both sexes no differences were observed in the time spent in the open and closed arms of the maze (Supplementary Fig. 4C, D). Notably, at baseline, neither female nor male *Cx3cr1*^CreER^:*Tnfrsf1b*^fl/fl^ mice showed abnormalities in grip strength and hind paw sensitivity compared to controls (Supplementary Fig. 4E–H). Collectively, these data suggest augmented exploratory behavior and mild anxiety-related traits in naïve female *Cx3cr1*^CreER^:*Tnfrsf1b*^fl/fl^ mice compared to *Tnfrsf1b*^fl/fl^ littermates, whereas no prominent differences between genotypes were found in male mice.

To evaluate the impact of microglial TNFR2 ablation on functional outcomes after pMCAO, we first performed a grip strength test at day 1 post-pMCAO to assess neuromuscular function and found no differences in total or side-specific forelimb strength between *Cx3cr1*^CreER^:*Tnfrsf1b*^fl/fl^ mice and *Tnfrsf1b*^fl/fl^ littermates of both sexes (Fig. 4A, B). In addition, no differences in hind paw pain sensitivity between genotypes were recorded with the Hargreaves test at day 3 and 5 post-pMCAO (Fig. 4C, D).

**Fig. 2.**
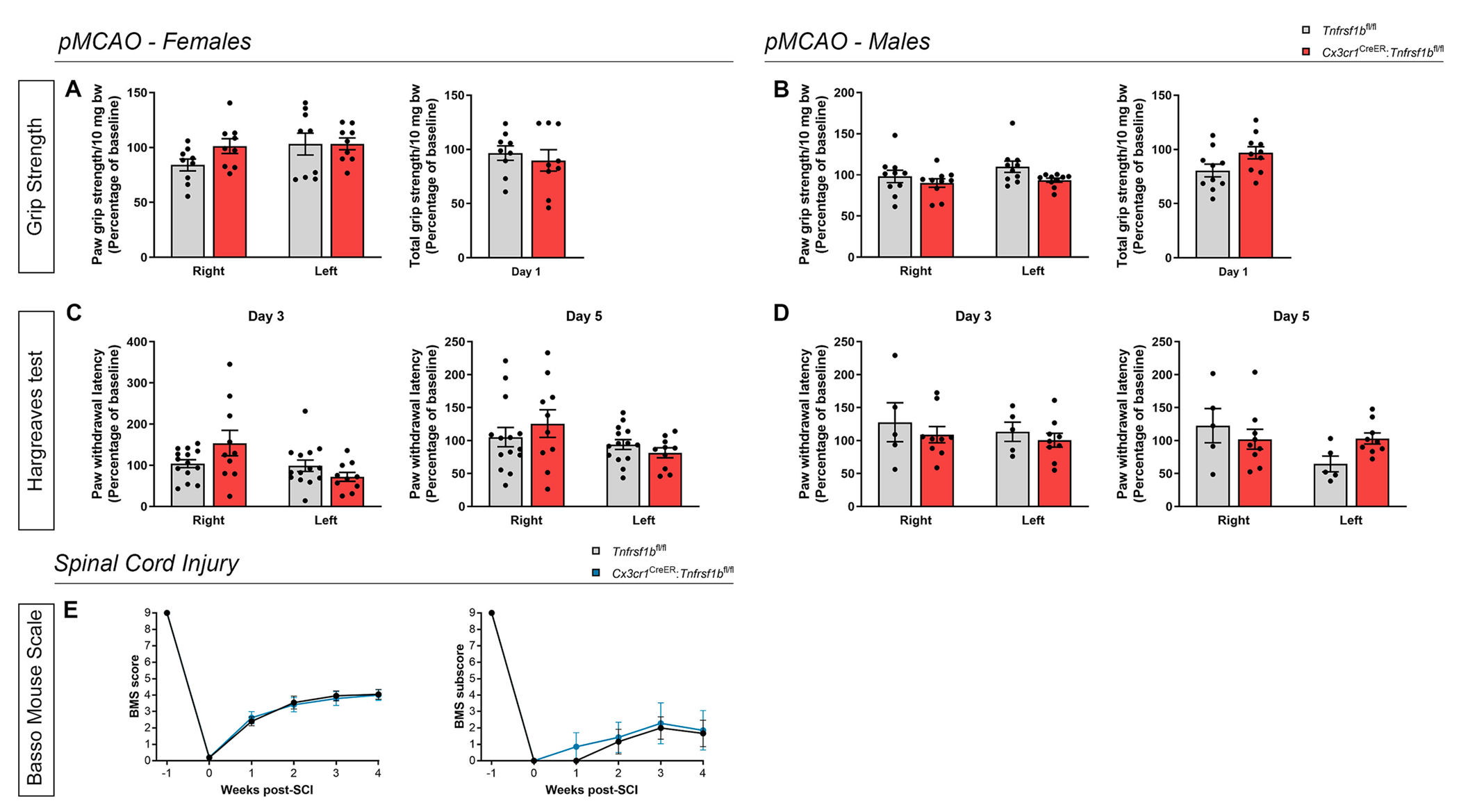
Conditional ablation of microglial TNFR2 does not affect functional measurements after ischemic stroke and spinal cord injury. (A-B) Quantification of side-specific and total grip strength in female (A) and male (B) *Cx3cr1*^CreER^:*Tnfrsf1b*^fl/fl^ mice and *Tnfrsf1b*^fl/fl^ littermates at day 1 post-pMCAO (females: n = 9/group; males: n = 10/group). (C-D) Quantification of right and left paw withdrawal latency during the Hargreaves test in female (C) and male (D) *Cx3cr1*^CreER^:*Tnfrsf1b*^fl/fl^ mice and *Tnfrsf1b*^fl/fl^ littermates at day 3 and 5 post-pMCAO (females: n = 10–14/group; males: n = 5–9/group). (E) Quantification of the clinical score and subscore according to the Basso mouse scale (BMS) in female *Cx3cr1*^CreER^:*Tnfrsf1b*^fl/fl^ mice and *Tnfrsf1b*^fl/fl^ littermates over time after SCI (score: n = 16–17/group; subscore: n = 6–7/group), bw: body weight.

In SCI, *Cx3cr1*^CreER^:*Tnfrsf1b*^fl/fl^ female mice displayed locomotor dysfunctions similar to those assessed for *Tnfrsf1b*^fl/fl^ littermates, as shown by the overlapping profiles of the BMS scores and subscores (Fig. 2E).

Taken together, these results suggest that microglial TNFR2 ablation, despite limiting the lesion volume in both pMCAO and SCI, does not impact behavioral outcomes after injury.

### 3.3. Ablation of microglial TNFR2 reduces pro-inflammatory cytokine levels after ischemic stroke

**Fig. 3.**
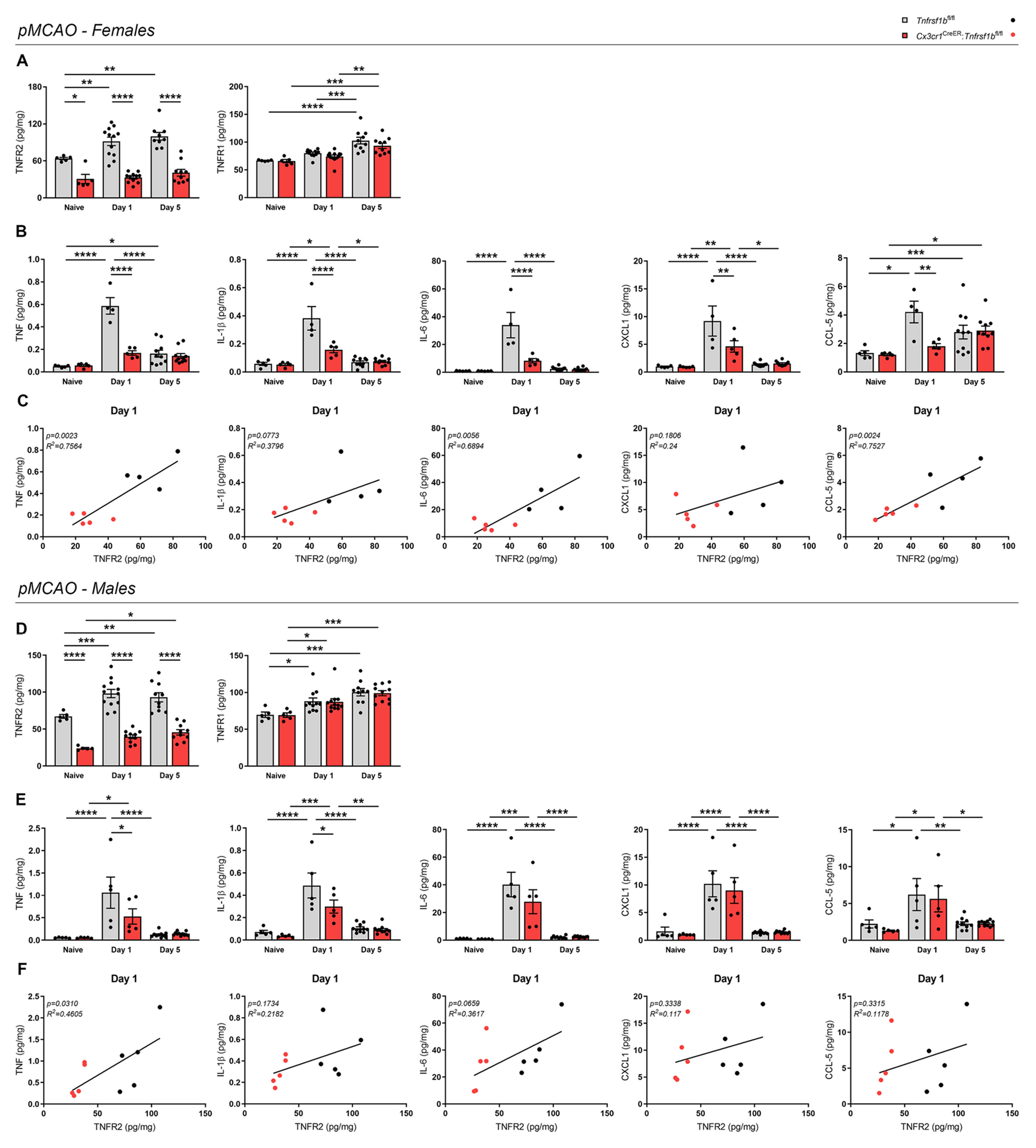
Conditional ablation of microglial TNFR2 reduces the protein levels of pro-inflammatory cytokines after ischemic stroke. (A) Quantification of TNFR2 and TNFR1 protein levels in the brain of female *Cx3cr1*^CreER^:*Tnfrsf1b*^fl/fl^ mice and *Tnfrsf1b*^fl/fl^ littermates in naïve conditions and at day 1 and 5 post-pMCAO (n = 5–12/group). * p < 0.05, ** p < 0.01, *** p < 0.001, **** p < 0.0001; Two-way ANOVA (TNFR2: Interaction^ns^, Time**, Genotype****; TNFR1: Interaction^ns^, Time****, Genotype^ns^) followed by Bonferroni’s multiple comparisons test. (B) Quantification of TNF, IL-1β, IL-6, CXCL1, and CCL-5 protein levels in the brain of female *Cx3cr1*^CreER^:*Tnfrsf1b*^fl/fl^ mice and *Tnfrsf1b*^fl/fl^ littermates in naïve conditions and at day 1 and 5 post-pMCAO (n = 5–12/group). * p < 0.05, ** p < 0.01, *** p < 0.001, **** p < 0.0001; Two-way ANOVA (TNF: Interaction****, Time****, Genotype****; IL-1β: Interaction****, Time****, Genotype***; IL-6: Interaction****, Time****, Genotype****; CXCL1: Interaction**, Time***, Genotype*; CCL-5: Interaction*, Time***, Genotype*) followed by Bonferroni’s multiple comparisons test. (C) Scatterplot representation of the linear correlation between TNFR2 (x axis) and TNF, IL-1β, IL-6, CXCL1, CCL-5 protein levels (y axis) in the brain of female *Cx3cr1*^CreER^:*Tnfrsf1b*^fl/fl^ mice and *Tnfrsf1b*^fl/fl^ littermates at day 1 post-pMCAO (n = 4–5/group). For correlation analysis, two-tailed Pearson test was used. (D) Quantification of TNFR2 and TNFR1 protein levels in the brain of male *Cx3cr1*^CreER^:*Tnfrsf1b*^fl/fl^ mice and *Tnfrsf1b*^fl/fl^ littermates in naïve conditions and at day 1 and 5 post-pMCAO (n = 5–11/group). * p < 0.05, ** p < 0.01, *** p < 0.001, **** p < 0.0001; Two-way ANOVA (TNFR2: Interaction^ns^, Time***, Genotype****; TNFR1: Interaction^ns^, Time****, Genotype^ns^) followed by Bonferroni’s multiple comparisons test. (E) Quantification of TNF, IL-1β, IL-6, CXCL1, and CCL-5 protein levels in the brain of male *Cx3cr1*^CreER^:*Tnfrsf1b*^fl/fl^ mice and *Tnfrsf1b*^fl/fl^ littermates in naïve conditions and at day 1 and 5 post-pMCAO (n = 5–11/group). * p < 0.05, ** p < 0.01, *** p < 0.001, **** p < 0.0001; Two-way ANOVA (TNF: Interaction^ns^, Time****, Genotype^ns^; IL-1β: Interaction^ns^, Time****, Genotype*; IL-6: Interaction^ns^, Time****, Genotype^ns^; CXCL1: Interaction^ns^, Time****, Genotype^ns^; CCL-5: Interaction^ns^, Time***, Genotype^ns^) followed by Bonferroni’s multiple comparisons test. (F) Scatterplot representation of the linear correlation between TNFR2 (x axis) and TNF, IL-1β, IL-6, CXCL1, CCL-5 protein levels (y axis) in the brain of male *Cx3cr1*^CreER^:*Tnfrsf1b*^fl/fl^ mice and *Tnfrsf1b*^fl/fl^ littermates at day 1 post-pMCAO (n = 5). For correlation analysis, two-tailed Pearson test was used.

To investigate whether ablation of microglial TNFR2 affected the inflammatory response after pMCAO, we first assessed the expression of TNF receptors and inflammatory mediators in the brain at day 1 and 5 post-pMCAO by multiplex electrochemiluminescence assay (Fig. 3 and Supplementary Fig. 5).

In females, TNFR2 was upregulated as a result of pMCAO (both at day 1 and day 5) in *Tnfrsf1b*^fl/fl^ littermates, contrary to *Cx3cr1*^CTeER^:*Tnfrsf1b*^fl/fl^ mice where TNFR2 expression remained comparable to naïve levels (Fig. 3A). This suggests that TNFR2 upregulation after pMCAO is primarily occurring in microglia. TNFR1 increased at day 5 post-pMCAO, with no significant differences between genotypes (Fig. 3A). Cerebral ischemia induced a transient increase of TNF, IL-1β, IL-6, CXCL1, and CCL5 at day 1 post-pMCAO, corresponding to the peak of pro-inflammatory cytokines typically observed in this model (Lambertsen et al., 2012a). Brain levels then returned to basal conditions by day 5 post-pMCAO for all molecules except for CCL5, which remained slightly elevated (Fig. 3B). Noteworthy, all were significantly reduced in microglial TNFR2-ablated female mice compared to controls at day 1 post-pMCAO (Fig. 3B), suggesting that microglial TNFR2 ablation may delay or suppress the acute inflammatory response to IS. Moreover, a positive linear correlation was identified at day 1 post-pMCAO between levels of TNFR2 and those of TNF, IL-1β, IL-6, and CCL5, but not CXCL1 (Fig. 3C), suggesting that in female mice cytokine production may be related to the presence of functional TNFR2 signaling in microglia. No significant changes between genotypes were found for IL-2, IL-4, IL-5, IL-10, IL-12p70, and IFNγ in female mice (Supplementary Fig. 5A).

In males, TNFR2 was upregulated as a result of pMCAO (both at day 1 and day 5) in *Tnfrsf1b*^fl/fl^ littermates, while in *Cx3cr1*^CreER^:*Tnfrsf1b*^fl/fl^ mice TNFR2 upregulation could be detected only at day 5 post-pMCAO (Fig. 3D). Notably, TNFR2 expression in *Cx3cr1*^CreER^:*Tnfrsf1b*^fl/fl^ mice was significantly lower than in *Tnfrsf1b*^fl/fl^ littermates at all time points considered (Fig. 3D). In addition, TNFR1 levels were not affected by microglial TNFR2 ablation (Fig. 3D). As for pro-inflammatory cytokines, in both genotypes a transient upregulation of TNF, IL-1β, IL-6, CXCL1, and CCL5 was detected at day 1 post-pMCAO. (Fig. 3E). Nevertheless, only TNF and IL-1β were found to be significantly reduced after microglial TNFR2 ablation in males (Fig. 3E), suggesting that microglial TNFR2 may be more relevant for cytokine production in females than in males. In line with these findings, in male mice a linear correlation was observed only between TNFR2 and TNF (Fig. 3F). No significant changes between genotypes were found in the other cytokines analyzed except IL-4 and IL-10, which were significantly reduced in naïve TNFR2-deficient mice compared to controls (Supplementary Fig. 5B).

Globally, these data indicate that microglial TNFR2 ablation limits the production of specific pro-inflammatory cytokines and chemokines after pMCAO, with mild sex-dependent differences.

### 3.4. Ablation of microglial TNFR2 has opposite sex-specific effects on leukocyte populations after ischemic stroke and spinal cord injury

**Fig. 4.**
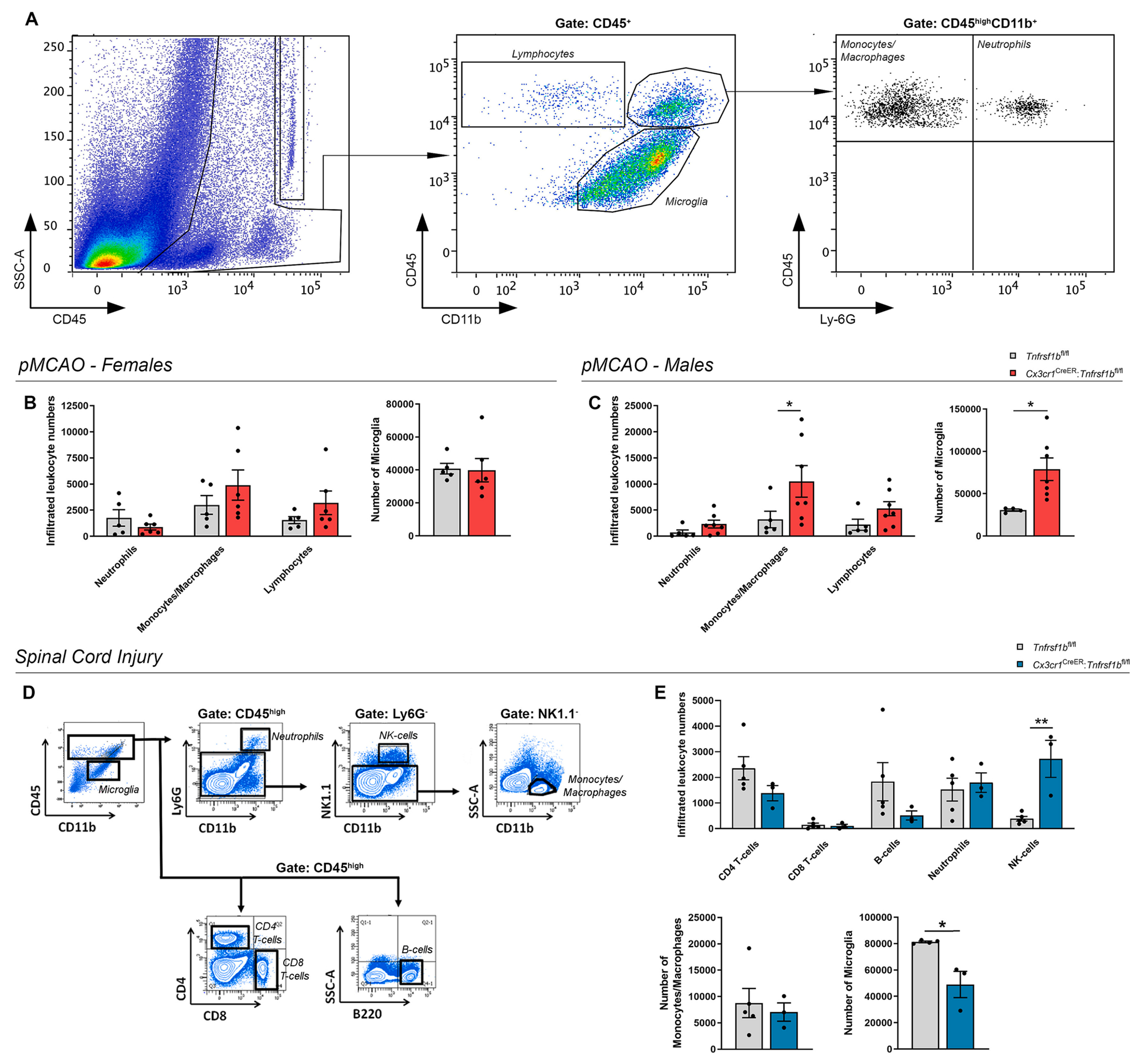
Conditional ablation of microglial TNFR2 has a sex-specific impact on microglial and leukocyte populations after IS and SCI. (A) Flow cytometry plots showing gating strategies for microglia and leukocyte analysis in the ipsilateral cortex at day 5 post-pMCAO. (B-C) Flow cytometry analysis of infiltrating immune cell populations and microglia in the ipsilateral cortex of female (B) and male (C) *Cx3cr1*^CreER^:*Tnfrsf1b*^fl/fl^ mice and *Tnfrsf1b*^fl/fl^ littermates at day 5 post-pMCAO (n = 5–6/group females, n = 5–7/group males). * p < 0.05, Student’s *t*-test. (D) Flow cytometry plots showing gating strategies for microglia and leukocyte analysis in the spinal cord at day 7 post-SCI. (E) Flow cytometry analysis of infiltrating immune cell populations and microglia in the spinal cord of female *Cx3cr1*^CreER^:*Tnfrsf1b*^fl/fl^ mice and *Tnfrsf1b*^fl/fl^ littermates at day 7 post-SCI (n = 3–4/group). * p < 0.05, ** p < 0.01; Student’s *t*-test.

After CNS injury, microglia-derived cytokines and chemokines facilitate the recruitment of circulating immune cells within the lesion, which in turn promote secondary damage (Iadecola and Anrather, 2011). To investigate the potential impact of microglial TNFR2 ablation on microglia and leukocyte populations, flow cytometry analysis was performed on *Cx3cr1*^CreER^:*Tnfrsf1b*^fl/fl^ and *Tnfrsf1b*^fl/fl^ mice at day 5 post-pMCAO and day 7 post-SCI (Fig. 4). In female mice subjected to pMCAO, ablation of microglial TNFR2 had no effect on the numbers of infiltrating lymphoid cells, monocytes/macrophages, and neutrophils in the ipsilateral cortex, whereas in males it induced a significant increase of monocytes/macrophages (Fig. 4A–C). Interestingly, the total number of microglial cells was significantly increased in male *Cx3cr1*^CreER^:*Tnfrsf1b*^fl/fl^ mice after pMCAO compared to controls, while no effects were found in females (Fig. 4A–C). In microglial TNFR2-ablated female mice subjected to SCI, no differences were found in the number of leukocytes infiltrating the injured spinal cord except for NK cells, that were significantly increased in *Cx3cr1*^CreER^:*Tnfrsf1b*^fl/fl^ compared to *Tnfrsf1b*^fl/fl^ mice (Fig. 4D, E). Moreover, lack of microglial TNFR2 significantly reduced the total number of microglia after SCI (Fig. 4D, E). Therefore, ablation of microglial TNFR2 had disease and sex-specific opposite effects on leukocyte infiltration and microglial cell number after pMCAO and SCI.

### 3.5. Ablation of microglial TNFR2 affects microglia responses after ischemic stroke and spinal cord injury in an opposite sex-specific manner

**Fig. 5.**
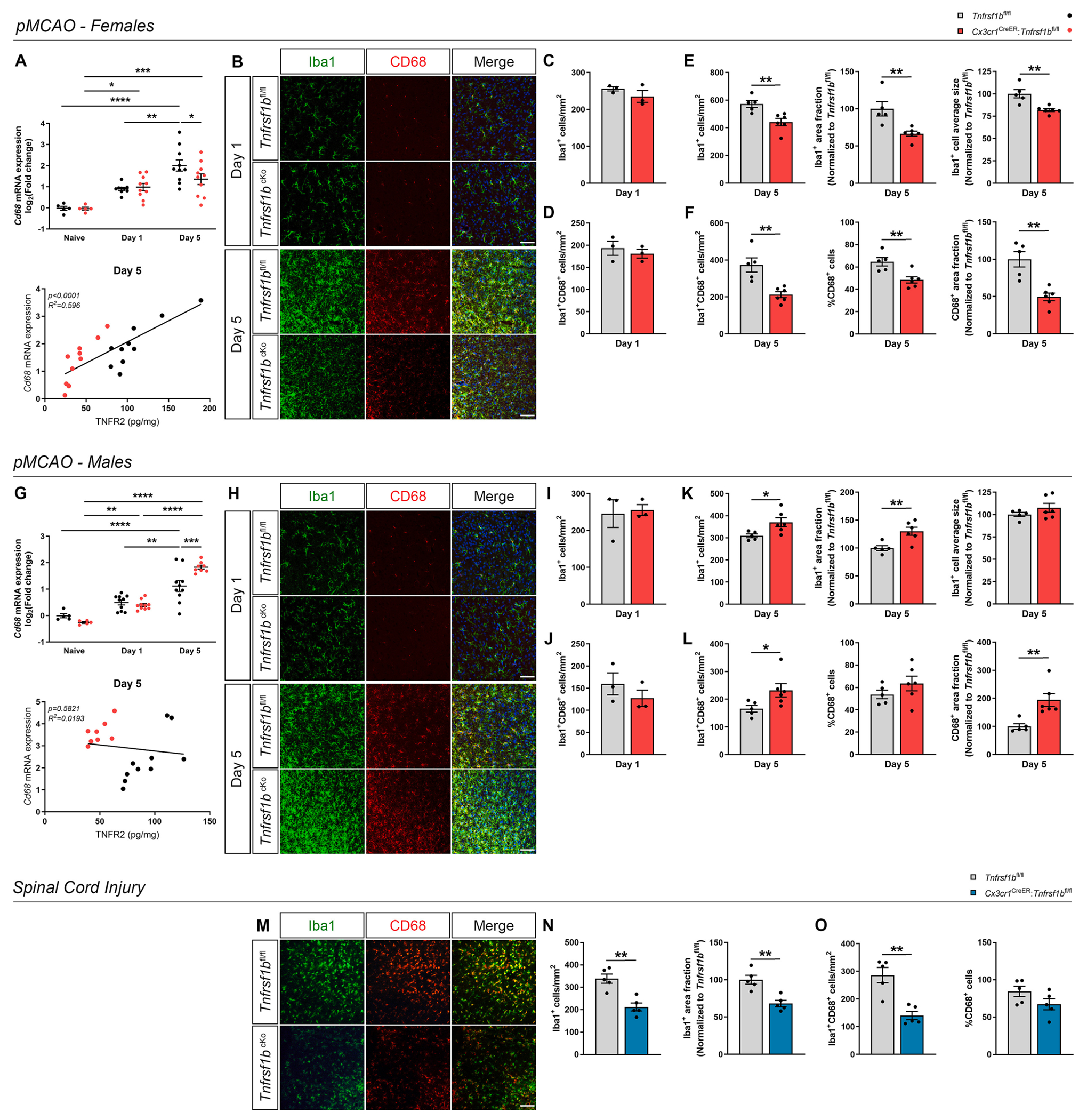
Conditional ablation of microglial TNFR2 affects microglial activation after ischemic stroke and spinal cord injury. (A) Quantification of *Cd68* mRNA expression in the brain of female *Cx3cr1*^CreER^:*Tnfrsf1b*^fl/fl^ mice and *Tnfrsf1b*^fl/fl^ littermates in naïve conditions and at day 1 and 5 post-pMCAO (n = 5–10/group) and scatterplot representation of the linear correlation between TNFR2 protein levels (x axis) and *Cd68* mRNA expression (y axis) at day 5 post-pMCAO (n = 10/group). * p < 0.05, ** p < 0.01, *** p < 0.001, **** p < 0.0001; Two-way ANOVA (Interaction^ns^, Time****, Genotype^ns^) followed by Bonferroni’s multiple comparisons test. For correlation analysis, two-tailed Pearson test was used. (B) Representative images of cells stained for Iba1 and CD68 at the boundary of the ischemic lesion (0–500 μm) in female *Cx3cr1*^CreER^:*Tnfrsf1b*^fl/fl^ (*Tnfrsf1b*^cKo^) mice and *Tnfrsf1b*^fl/fl^ littermates at day 1 and 5 post-pMCAO. Scale bar: 50 μm. (C-D) Quantification of Iba1^+^ (C) and Iba1^+^CD68^+^ cell density (D) at the boundary of the ischemic lesion (0–500 μm) in female *Cx3cr1*^CreER^:*Tnfrsf1b*^fl/fl^ mice and *Tnfrsf1b*^fl/fl^ littermates at day 1 post-pMCAO (n = 3/group). (E-F) Quantification of Iba1^+^ cell density, Iba1^+^ area fraction, Iba1^+^ average cell size (E), and of Iba1^+^CD68^+^ cell density, percentage of CD68^+^ cells, CD68^+^ area fraction (F) at the boundary of the ischemic lesion (0–500 μm) in female *Cx3cr1*^CreER^:*Tnfrsf1b*^fl/fl^ mice and *Tnfrsf1b*^fl/fl^ littermates at day 5 post-pMCAO (n = 5–6/group). ** p < 0.01; Student’s *t*-test. (G) Quantification of *Cd68* mRNA expression in the brain of male *Cx3cr1*^CreER^:*Tnfrsf1b*^fl/fl^ mice and *Tnfrsf1b*^fl/fl^ littermates in naïve conditions and at day 1 and 5 post-pMCAO (n = 5–10/group) and scatterplot representation of the linear correlation between TNFR2 protein levels (x axis) and *Cd68* mRNA expression (y axis) at day 5 post-pMCAO (n = 10/group). * p < 0.05, ** p < 0.01, *** p < 0.001, **** p < 0.0001; Two-way ANOVA (Interaction***, Time****, Genotype^ns^) followed by Bonferroni’s multiple comparisons test. For correlation analysis, two-tailed Pearson test was used. (H) Representative images of cells stained for Iba1 and CD68 at the boundary of the ischemic lesion (0–500 μm) in male *Cx3cr1*^CreER^:*Tnfrsf1b*^fl/fl^ (*Tnfrsf1b*^cKo^) mice and *Tnfrsf1b*^fl/fl^ littermates at day 1 and 5 post-pMCAO. Scale bar: 50 μm. (I-J) Quantification of Iba1^+^ (I) and Iba1^+^CD68^+^ cell density (J) at the boundary of the ischemic lesion (0–500 μm) in male *Cx3cr1*^CreER^:Tnfrsf1b^fl/fl^ mice and *Tnfrsf1b*^fl/fl^ littermates at day 1 post-pMCAO (n = 3/group). (K-L) Quantification of Iba1^+^ cell density, Iba1^+^ area fraction, Iba1^+^ average cell size (K), and of Iba1^+^CD68^+^ cell density, percentage of CD68^+^ cells, CD68^+^ area fraction (L) at the boundary of the ischemic lesion (0–500 μm) in male *Cx3cr1*^CreER^:*Tnfrsf1b*^fl/fl^ mice and *Tnfrsf1b*^fl/fl^ littermates at day 5 post-pMCAO (n = 6/group). * p < 0.05, ** p < 0.01; Student’s *t*-test. (M) Representative images of cells stained for Iba1 and CD68 in the *peri*-lesion area of female *Cx3cr1*^CreER^:*Tnfrsf1b*^fl/fl^ (*Tnfrsf1b*^cKo^) mice and *Tnfrsf1b*^fl/fl^ littermates at day 28 post-SCI. Scale bar: 50 μm. (N-O) Quantification of Iba1^+^ cell density, Iba1^+^ area fraction (N), and of Iba1^+^CD68^+^ cell density, percentage of CD68^+^ cells (O) in the *peri*-lesion area of female *Cx3cr1*^CreER^:*Tnfrsf1b*^fl/fl^ mice and *Tnfrsf1b*^fl/fl^ littermates at day 28 post-SCI (n = 5/group). ** p < 0.01; Student’s *t*-test.

To test whether TNFR2 ablation affected the reactive state of microglia and macrophages after IS, we next performed qPCR and immunohistochemical analyses (Fig. 5). First, we evaluated gene expression of the scavenger receptor *Cd68,* an established marker of microglia and macrophage reactivity (Perego et al., 2011), in the brain in naïve conditions and at day 1 and 5 post-pMCAO. Interestingly, *Cd68* expression was significantly reduced in female microglial TNFR2-ablated mice compared to controls at day 5 post-pMCAO (Fig. 5A), whereas it was upregulated in male TNFR2-ablated mice (Fig. 5G). Moreover, a positive linear correlation was observed between *Cd68* mRNA expression and TNFR2 protein levels in female but not in male mice at day 5 post-pMCAO (Fig. 5A, G), suggesting a sex-dependent role of TNFR2 in driving microglial responses to ischemic damage.

To gain further insight on microglial activation at the boundaries of the ischemic lesion, double immunolabeling for Iba1 and CD68 was performed on brain sections collected from female and male *Cx3cr1*^CreER^:*Tnfrsf1b*^fl/fl^ and *Tnfrsf1b*^fl/fl^ mice at day 1 and 5 post-pMCAO (Fig. 5B, H). We focused on the region within 500 μm from the lesion border, in which Iba1^+^ microglia characterized by a more ramified morphology are prevalent. Rounded monocyte/macrophage-like cells which localize within the lesion core are not included in this analysis (Raffaele et al., 2021). At day 1 post-pMCAO, no significant differences in the number of Iba1^+^ and Iba1^+^CD68^+^ cells were observed between genotypes both in females and males (Fig. 5C, D, I, J). Instead, at day 5 post-pMCAO, the density of Iba1^+^ cells, the area they occupied, and their average size were significantly reduced in female *Cx3cr1*^CreER^:*Tnfrsf1b*^fl/fl^ mice compared to controls (Fig. 5E). Moreover, *Cx3cr1*^CreER^:*Tnfrsf1b*^fl/fl^ female mice displayed a significant reduction in the density of Iba1^+^CD68^+^ cells, percentage of CD68^+^ cells within the total Iba1^+^ microglial population, and area occupied by CD68 staining (Fig. 5F). Contrary to females, in male mice microglial TNFR2 ablation resulted in a significant increase in density and total area of Iba1^+^ cells (Fig. 5K), as well as density of Iba1^+^CD68^+^ cells and CD68^+^ area fraction at day 5 post-pMCAO (Fig. 5L). Noteworthy, immunohistochemical analysis also confirmed reduced TNFR2 expression in Iba1^+^ microglia-like cells surrounding the ischemic lesion in *Cx3cr1*^CreER^:*Tnfrsf1b*^fl/fl^ mice compared to controls (Supplementary Fig. 6A).

A similar analysis was performed in female mice subjected to SCI, revealing a significant reduction in Iba1^+^ cell density, Iba1^+^ area fraction, and Iba1^+^CD68^+^ cell density in the *peri*-lesion area of *Cx3cr1*^CreER^:*Tnfrsf1b*^fl/fl^ mice at 28 days post-SCI, but not in the percentage of CD68^+^ cells (Fig. 5M–O).

Collectively, our data indicate that ablation of TNFR2 has opposite sex-dependent effects on microglial reactive state in the immediate surroundings of ischemic and spinal cord lesions, which was found to be limited in female and enhanced in male conditional knockout mice. No obvious sex-dependent differences could be detected in *Tnfrsf1b* gene expression assessed in purified microglia from the ipsilateral cortex of male and female *Cx3cr1*^CreER^:*Tnfrsf1b*^fl/fl^ mice and sex-matched controls at day 5 post-pMCAO, likely excluding the opposite effects observed be due to different microglial TNFR2 levels (Supplementary Fig. 6B).

### 3.6. TNFR2 levels are elevated in the CSF of individuals with ischemic stroke and spinal cord injury

Due to its direct contact with the CNS tissue, the CSF mirrors the biochemical processes taking place in the injured brain and spinal cord, but data regarding TNFR2 levels in the CSF of IS and SCI human cases are still limited.

**Fig. 6.**
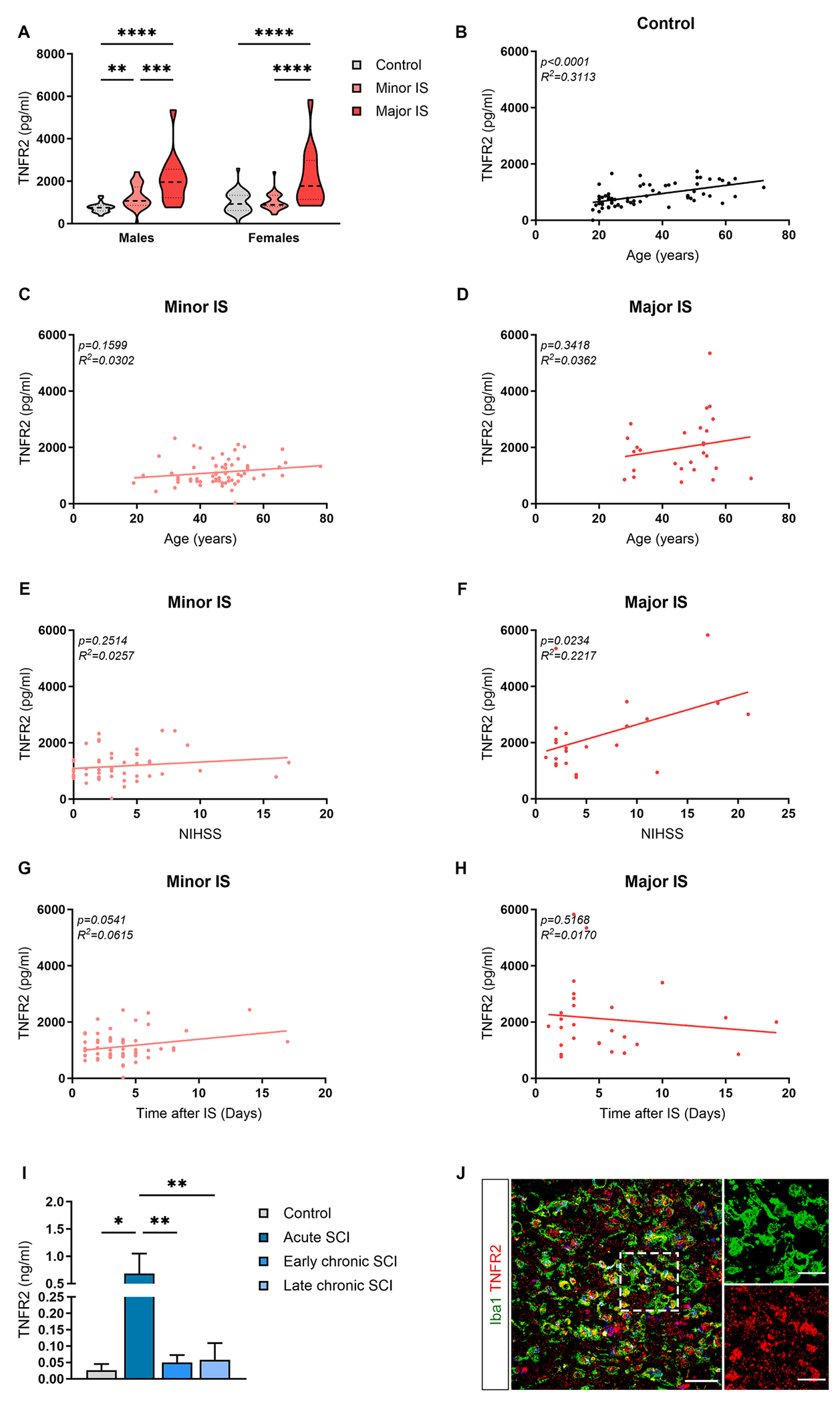
TNFR2 levels are increased in the cerebrospinal fluid of patients affected by ischemic stroke and spinal cord injury. (A) Quantification of TNFR2 protein levels in the cerebrospinal fluid (CSF) of male and female healthy controls (n = 25–42) and patients with minor (n = 37–31) and major (n = 14–14) ischemic stroke (IS). ** p < 0.01, *** p < 0.001, **** p < 0.0001; Two-way ANOVA (Interaction^ns^, Sex^ns^, Severity****) followed by Sidak’s multiple comparison test. (B-D) Scatterplot representation of the linear correlation between age (x axis) and TNFR2 protein levels (y axis) in the CSF of sex-mixed healthy controls (B, n = 66) and patients with minor (C, n = 67) and major (D, n = 27) IS. For correlation analysis, two-tailed Pearson test was used. (E-F) Scatterplot representation of the linear correlation between National Institute of Health Stroke Scale (NIHSS, x axis) and TNFR2 protein levels (y axis) in the CSF of sex-mixed patients with minor (E, n = 53) and major (F, n = 23) IS. For correlation analysis, two-tailed Pearson test was used. (G-H) Scatterplot representation of the linear correlation between time after IS (x axis) and TNFR2 protein levels (y axis) in the CSF of sex-mixed patients with minor (G, n = 61) and major (H, n = 27) IS. For correlation analysis, two-tailed Pearson test was used. (I) Quantification of TNFR2 protein levels in the CSF of healthy controls (n = 5) and patients with acute (n = 5), early chronic (n = 12), and late chronic (n = 11) spinal cord injury (SCI). ** p < 0.01; One-way ANOVA followed by Tukey’s multiple comparison test. (J) Representative image of cells stained for Iba1 and TNFR2 at the boundary of the ischemic lesion (0–500 μm) in a human stroke case. Scale bar: 50 μm. The enlargement shows cells double positive for Iba1 and TNFR2. Scale bar: 25 μm.

To fill this gap, we quantified TNFR2 protein levels in the CSF of individuals affected by IS and healthy controls. Male subjects with minor or major IS had a significant increase in TNFR2 levels compared to healthy controls (Fig. 6A). Moreover, TNFR2 was significantly higher in the CSF of male individuals with major versus minor IS (Fig. 6A). In females, a significant increase of TNFR2 could only be detected after major IS compared to controls and minor IS cases (Fig. 6A). No significant differences were detected in male versus female subjects (Fig. 6A).

Interestingly, a positive linear correlation was observed between TNFR2 levels in the CSF and age of healthy subjects (Fig. 6B), while no correlation was found for minor and major IS (Fig 6C, D). Moreover, we observed a positive correlation between TNFR2 and disease severity, assessed by the National Institute of Health Stroke Scale (NIHSS), in major but not minor IS cases (Fig. 6E, F). No significant correlation was found between TNFR2 levels and the time of CSF sample collection after IS (Fig. 6G, H).

In individuals affected by traumatic SCI, TNFR2 levels increased at the acute stage of SCI compared to both healthy controls and participants at the early and late chronic phase after injury (Fig. 6I). These results suggest that TNFR2 protein levels in the CSF are similarly increased after IS and SCI, possibly reflecting the extent of neuroinflammation at the lesion site.

Microglia are the first cells responding to injury in the brain and spinal cord by acquiring multiple functional states contributing to damage evolution and repair mechanisms (Zrzavy et al., 2018; Zrzavy et al., 2021). Thus, we evaluated microglial TNFR2 expression in tissue sections from human IS by immunohistochemistry, revealing extensive TNFR2 co-localization with Iba1^+^ reactive microglial cells at lesion boundaries, characterized by a typical ameboid shape with enlarged cell bodies and reduced arborization (Fig. 6j). On this basis, the changes in TNFR2 levels detected in the CSF of individuals with IS and SCI may reflect the dynamics of microglial activation in these neuropathological conditions.

## 4. Discussion

It is widely accepted that TNF plays a key role in the inflammatory response after IS and SCI (Lambertsen et al., 2018; Lund et al., 2022a). TNF-targeted therapies, however, have largely failed in the treatment of neurological diseases, possibly due to the often-antithetic functions of the two TNF receptors and, importantly, to the limited knowledge regarding the exact contribution of microglial TNFR2 signaling after CNS injury. In the present study, we demonstrate a pivotal and sex-dependent role of microglial TNFR2 in shaping the neuroinflammatory environment after IS and SCI by affecting cytokine levels, leukocyte infiltration, and microglial reactivity at lesion boundaries. Moreover, we show that TNFR2 is expressed in reactive microglial cells surrounding human ischemic lesions, and that increased TNFR2 levels in the CSF of human IS and SCI cases correlate with disease stage and severity, possibly reflecting the extent of the microglial response at the lesion site.

### 4.1. Microglial TNFR2 signaling has an opposite sex-specific role in the early inflammatory response after acute CNS injury

Here, we showed that microglial TNFR2 ablation leads to a significant reduction in lesion volume after IS and SCI. This, however, did not result in improved functional outcome as assessed by grip strength, Hargreaves, and BMS tests. These data are in agreement with several seminal reports indicating that in patients, standard measurements, such as lesion volume, alone have limited prognostic value with respect to functional deficit type, severity, and recovery (Ernst et al., 2017; Fouad et al., 2021; Lyden et al., 1997; Vora et al., 2011). In addition, this agrees with studies demonstrating that in patients with small ischemic stroke lesions, no relationship between lesion volumes and functional status was seen (Schiemanck et al., 2005). More accurate evaluations, including functional MRI and electrophysiological measurements, are thus necessary to properly assess the impact of microglial TNFR2 on functional parameters. It is worth mentioning that, based on the peculiar temporal dynamics of the inflammatory response in the two CNS injury models (Raffaele et al., 2021; Lund et al., 2022: PMID: 35604578), the lesion size assessment performed in this study may reflect different disease stages, i.e., acute in pMCAO versus chronic in SCI. Increasing the harmonization within different CNS injury models in terms of readout evaluations may improve the translation of research findings obtained in one experimental setting to different pathological conditions.

We also observed a significant decrease in cytokine (TNF, IL-6, IL-1β) and chemokine (CXCL1 and CCL5) levels in the brain of TNFR2-ablated mice during the hyperacute phase after IS, corresponding to the first 24 h after injury, suggesting that cytokine production by microglia, at least partly, relies on functional TNFR2 signaling. This is also supported by the positive correlation observed between the protein levels of TNFR2 and pro-inflammatory cytokines in female mice. Conversely, microglial TNFR2 ablation had a limited impact on inflammatory cytokines in males. These data initially suggested a sexual dimorphism in TNFR2-dependent inflammatory responses after CNS injury, similarly to what observed for other cytokine receptors (Niu et al., 2023; Tariq et al., 2022). We also found that the number of leukocytes infiltrating the injured cerebral cortex and spinal cord was largely unaffected by microglial TNFR2 ablation in female animals, at least at the time points considered. On the other hand, monocyte/macrophage populations were found to be significantly increased after ischemic injury in males in the absence of microglial TNFR2, similarly to what we previously showed in the experimental autoimmune encephalomyelitis (EAE) model (Gao et al., 2017). Importantly, these data are in line with previous findings indicating that resident microglia are the prevalent immune cells at very early stages after CNS injury, rapidly adopting an inflammatory profile associated with cytokine secretion and recruitment of immune cells (Hernandez et al., 2023), which may be at least partly regulated by TNFR2 signaling.

Within the first week after ischemic injury, microglia shift from an inflammatory to a proliferative state, expanding their number and acquiring pro-regenerative traits required to initiate tissue remodeling (Hernandez et al., 2023; Raffaele and Fumagalli, 2022). In the present study, conditional ablation of microglial TNFR2 in female mice resulted in a significant reduction in the density and size of Iba1^+^ microglial cells recruited to lesion boundaries at early stages after IS and SCI, as shown by immunofluorescence and flow cytometry analyses, suggestive of a dampened microglial response to injury. Moreover, in both IS and SCI models, we found that ablation of microglial TNFR2 resulted in a significant decrease of Iba1^+^ cells co-expressing the scavenger receptor CD68 in the *peri*-lesion area. This was also reflected by a significant decrease in *Cd68* gene expression 5 days after pMCAO, with *Cd68* mRNA levels correlating with TNFR2 protein, suggesting that TNFR2 may contribute to the induction of CD68 in microglia. Since CD68 normally decorates reactive, phagocytic microglia (Lier et al., 2021), our data suggest that the clearance capacity of microglia in females may be reduced in the absence of TNFR2. This is in line with our previous report where expression of genes related to pathogen recognition (*Fcγr3*), phagocytosis (*Trem2*), and tissue surveillance (Siglec and purinergic receptors) was found to be reduced in TNFR2-ablated microglia isolated from the mouse spinal cord at the onset of EAE, and phagocytosis defects were observed in cultured TNFR2^−/−^ microglia (Gao et al., 2017). Surprisingly, we found the opposite effect in male mice, in which ablation of microglial TNFR2 resulted in increased density of microglial cells at ischemic lesion borders, and enhanced *Cd68* gene expression and colocalization with Iba1^+^ cells. Despite we were not able to confirm a similar impact of microglial TNFR2 ablation after SCI in males, which is a clear limitation of our study, these results open to the intriguing possibility that TNFR2 signaling may play a role in driving the sex-dependent differences in microglia reactivity described after stroke and other types of acute CNS injury (Banerjee and McCullough, 2022; Tariq et al., 2022; Ugidos et al., 2022). In our experiments, no obvious sex-dependent differences could be detected in microglial *Tnfrsf1b* expression levels after stroke, suggesting that the sexual dimorphism observed is not dependent on TNFR2 levels themselves, but it is likely due to different mechanisms. A possible explanation could be related to differences in downstream signaling pathways, which include the PI3K-PKB/Akt pathway that is pivotally involved in the immunomodulatory effects of TNFR2 (Dong et al., 2016; Fu et al., 2021). This hypothesis is supported by previous studies reporting discrepancies in PI3K-PKB/Akt signaling between males and females in different inflammatory conditions (Pinto-Benito et al., 2022; Robinson et al., 2022; Santiago et al., 2021). Accordingly, our own results showed that pMCAO-induced Akt phosphorylation was completely abolished in microglial TNFR2-ablated male mice, while it was reduced in females but remained significantly increased compared to naïve conditions. These data suggest that specific downstream signaling cascades, including the PI3K-PKB/Akt pathway, may be engaged upon TNFR2 stimulation in a different extent in males versus females, driving consequent sex-specific effects. However, we do not exclude that additional mechanisms may contribute to the sex-specific effects of microglial TNFR2 ablation, including a different epigenetic regulation (i.e., DNA methylation, histone modifications, and non-coding RNAs) of TNFR2 downstream genes in males and females, as already shown for interferon-responsive genes in rodent models and human stroke cases (Qi et al., 2021; Ratnu et al., 2017).

One open question remains regarding the impact of TNFR2-dependent microglial responses on tissue repair processes and consequent long-term functional improvement after CNS injury. Indeed, while seminal studies point at microglia as drivers of inflammatory damage, accumulating evidence suggest that early microglia reactivity is crucial to promote axonal growth, myelin regeneration, and angiogenesis at lesion sites (Brennan et al., 2022; Jiang et al., 2020; Raffaele et al., 2021). Considering that TNFR2 signaling in microglia has been implicated in neuroprotection and remyelination (Probert, 2015; Gao et al., 2017), the apparently beneficial effects of microglial TNFR2 ablation on lesion size and inflammatory cytokine levels that we observed at early time points may limit the signals that are required to initiate the pro-regenerative response, hindering recovery at chronic stages after CNS injury. Further investigation in this direction will be crucial to clarify the effects of microglial TNFR2 signaling on CNS regeneration in males and females and to tailor novel precision medicine approaches to achieve efficient recovery in both sexes.

### 4.2. TNFR2 levels in the CSF of human IS and SCI cases reflect disease stage and severity

Therapeutic perspectives in IS and SCI may derive from the identification of novel biomarkers, allowing to improve diagnostic precision, predict clinical outcomes, and monitor disease progression as well as the efficacy of putative treatments (Rodrigues et al., 2018; Simpkins et al., 2020). In this respect, the CSF represents an ideal compartment to investigate the biochemical processes occurring in the CNS (Gaetani et al., 2020). Several CSF molecules have been proposed as reliable markers for glial cell activation within CNS, potentially reflecting the involvement of astrogliosis (i.e. GFAP) and microgliosis (i.e. soluble TREM2) in the pathogenesis of neurological disorders (Gaetani et al., 2020; Gao et al., 2023).

Here, we show that TNFR2 protein levels in the CSF are significantly increased in IS cases when compared to healthy subjects and allow to discriminate different types of IS subjects based on disease severity. According to the IS data, we also observed a significant increase of TNFR2 in the CSF of SCI cases at acute disease stage, which display ongoing microglia reactivity, as compared to both healthy controls and chronic stage subjects, characterized by a diminishing inflammatory response (Lund et al., 2022b). After IS patient stratification by sex, we found that a similar TNFR2 pattern is maintained in the CSF of male subjects with minor and major IS, while in females a significant increase of TNFR2 levels could be detected only in major IS cases but not in minor IS individuals. This sexual dimorphism could be due to the sex-related differences in the microglial response during stroke pathogenesis (Ugidos et al., 2022) and certainly deserves further investigation. Interestingly, we also observed a positive linear correlation between TNFR2 levels in the CSF and the age of healthy controls, but not minor and major IS subjects. This data suggests that TNFR2 levels may also be used to monitor the neuroinflammatory response during aging and to track the related cognitive decline, in line with a recently published cluster analysis (Peng et al., 2020). Hence, we propose that TNFR2 levels in the CSF may represent a useful biomarker reflecting the inflammatory processes taking place in IS and SCI patients, as already proposed in the context of other neurodegenerative diseases (Jiang et al., 2011; Magliozzi et al., 2021; Pillai et al., 2021).

In conclusion, our study advanced the general understanding of the role of TNFR2 signaling in regulating microglial responses after IS and SCI, setting the stage for novel therapeutic approaches to tackle these invalidating neurological diseases.

## Supplementary Material

Supplementary table

Supplementary figures

## Data Availability

Data will be made available on resonable request.
