# Supplementary material for: Microglial TNFR2 signaling regulates the inflammatory response after CNS injury in a sex-specific fashion
Source: Brain Behav Immun. Author manuscript; Available in PMC 2024 Oct 24. (PMC11500189; doi:10.1016/j.bbi.2023.12.025)
Supplement: Supplementary figures [file NIHMS2025583-supplement-Supplementary_figures.pdf]

Microglial TNFR2 signaling regulates the inflammatory response after CNS injury in a sex-specific fashion

Stefano Raffaele[1,2,†], Estrid Thougaard[1†], Cathrine C. H. Laursen[1,3,†], Han Gao[4,5,†], Katrine M. Andersen[1], Pernille V. Nielsen[1], Natalia Ortí-Casañ[6], Morten Blichfeldt-Eckhardt[7,8], Simon Koch[9], Milani Deb-Chatterji[9], Tim Magnus[9], Jane Stubbe[10], Kirsten Madsen[10], Morten Meyer[1,3,11], Matilda Degn[12], Ulrich L.M. Eisel[6], Agnieszka Wlodarczyk[1,3], Marta Fumagalli[2], Bettina H. Clausen[1,3], Roberta Brambilla[3,13,*], and Kate L. Lambertsen[1,3,11,*]

[1]Department of Neurobiology Research, Institute of Molecular Medicine, University of Southern Denmark, 5000 Odense C, Denmark.

[2]Department of Pharmacological and Biomolecular Sciences "Rodolfo Paoletti", Università degli Studi di Milano, 20133 Milan, Italy.

[3]Department of Clinical Research, BRIDGE-Brain Research Inter Disciplinary Guided Excellence, University of Southern Denmark, 5000 Odense C, Denmark.

[4]Department of Spine Surgery, The Third Affiliated Hospital of Sun Yat-Sen University, 510630 Guangzhou, China.

[5]Guangdong Provincial Center for Engineering and Technology Research of Minimally Invasive Spine Surgery, 510630 Guangzhou, China.

[6]Department of Molecular Neurobiology, Groningen Institute for Evolutionary Life Sciences, University of Groningen, Groningen 9713 AV, Netherlands.

[7]Department of Anaesthesiology, Vejle Hospital, 7100 Vejle, Denmark.

[8]Department of Clinical Research, University of Southern Denmark, 5000 Odense C, Denmark.

[9]Department of Neurology, University Medical Center Hamburg-Eppendorf, 20246 Hamburg, Germany.

[10]Department of Cardiovascular and Renal Research, Institute of Molecular Medicine, University of Southern Denmark, 5000 Odense C, Denmark.

[11]Department of Neurology, Odense University Hospital, 5000 Odense C, Denmark.

[12]Gubra, 2970 Hørsholm, Denmark.

[13]The Miami Project to Cure Paralysis, University of Miami Miller School of Medicine, Miami FL, USA.

[†] Joint first authors

- Co-corresponding authors: Kate Lykke Lambertsen, J. B. Winsloewsvej 21 st., 5000 Odense C, Denmark, tel: +45 6550 3806, email: klambertsen@health.sdu.dk & Roberta Brambilla, 1095 NW 14th Terrace #48, Miami, FL 33136, USA, tel: +1 305-243-6001, email: RBrambilla@med.miami.edu

SUPPLEMENTARY MATERIAL

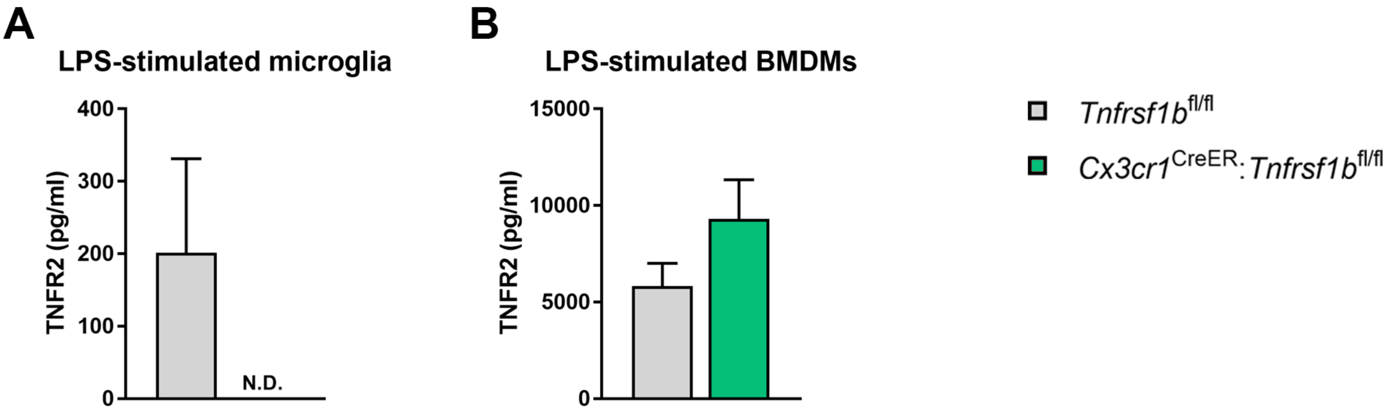

Supplementary Figure 1. (A-B) Quantification of TNFR2 protein levels in primary cultures of microglia (A) and bone marrow-derived macrophages (BMDMs, B) isolated from naïve *Cx3cr1*^{CreER}:*Tnfrsf1b*$^{\text{fl/fl}}$ mice and *Tnfrsf1b*$^{\text{fl/fl}}$ littermates and stimulated with lipopolysaccharide (LPS; n=3/group). N.D.=Non detectable.

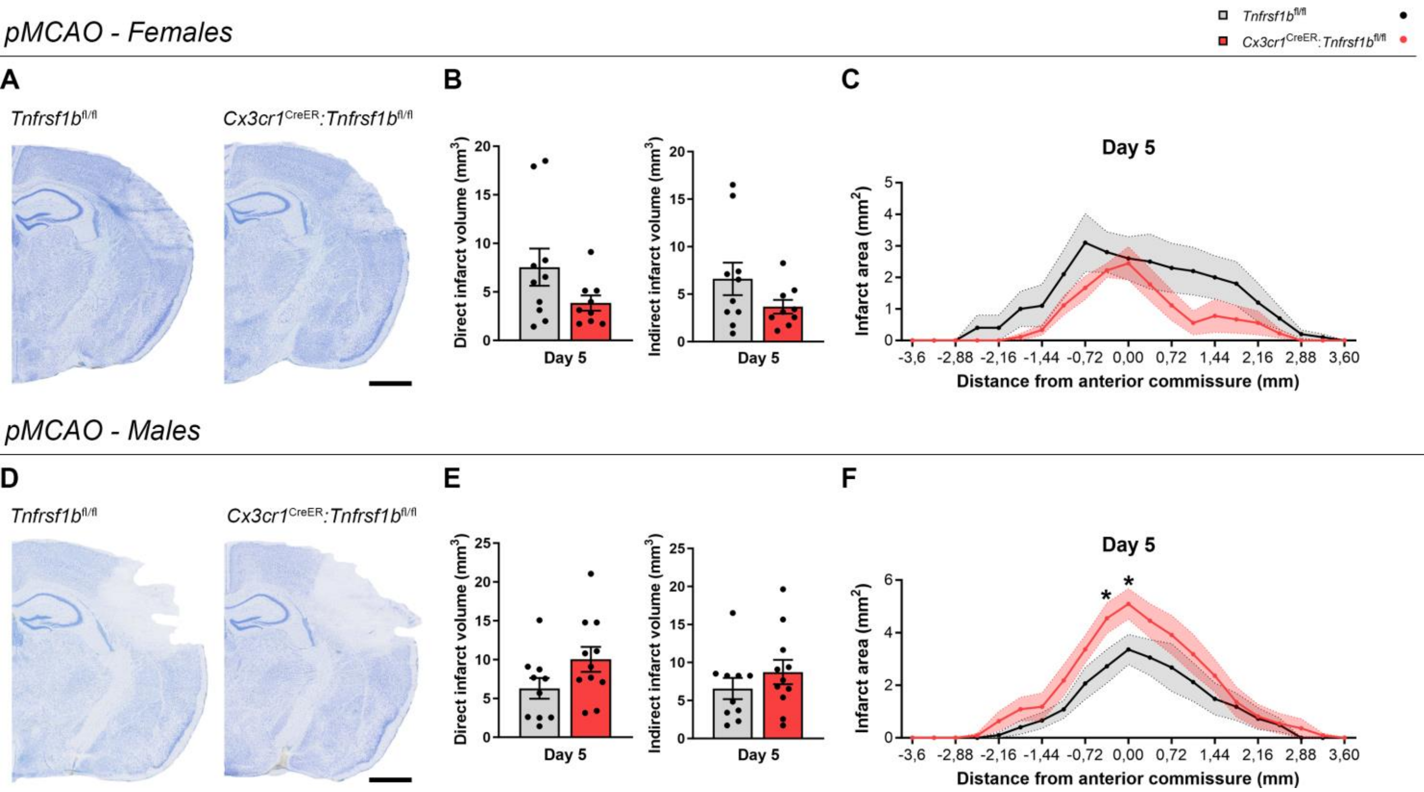

Supplementary Figure 2. (**A**) Representative images of toluidine blue-stained brain sections from female *Cx3cr1*^{CreER}:*Tnfrsf1b*$^{\text{fl/fl}}$ mice and *Tnfrsf1b*$^{\text{fl/fl}}$ littermates at day 5 post-permanent middle cerebral artery occlusion (pMCAO). Scalebar: 2,5 mm. (**B**) Quantification of the direct and indirect infarct volumes in female *Cx3cr1*^{CreER}:*Tnfrsf1b*$^{\text{fl/fl}}$ mice and *Tnfrsf1b*$^{\text{fl/fl}}$ littermates at day 5 post-pMCAO (n=9-10). (**C**) Rostrocaudal distribution of the ischemic infarct in female *Cx3cr1*^{CreER}:*Tnfrsf1b*$^{\text{fl/fl}}$ mice and *Tnfrsf1b*$^{\text{fl/fl}}$ littermates at day 5 post-pMCAO (n=9-10). (**D**) Representative images of toluidine blue-stained brain sections from male *Cx3cr1*^{CreER}:*Tnfrsf1b*$^{\text{fl/fl}}$ mice and *Tnfrsf1b*$^{\text{fl/fl}}$ littermates at day 5 post-pMCAO. Scalebar: 2,5 mm. (**E**) Quantification of the direct and indirect infarct volumes in male *Cx3cr1*^{CreER}:*Tnfrsf1b*$^{\text{fl/fl}}$ mice and *Tnfrsf1b*$^{\text{fl/fl}}$ littermates at day 5 post-pMCAO (n=10-11). (**F**) Rostrocaudal distribution of the ischemic infarct in male *Cx3cr1*^{CreER}:*Tnfrsf1b*$^{\text{fl/fl}}$ mice and *Tnfrsf1b*$^{\text{fl/fl}}$ littermates at day 5 post-pMCAO (n=10-11). * $p<0.05$; Multiple t-test.

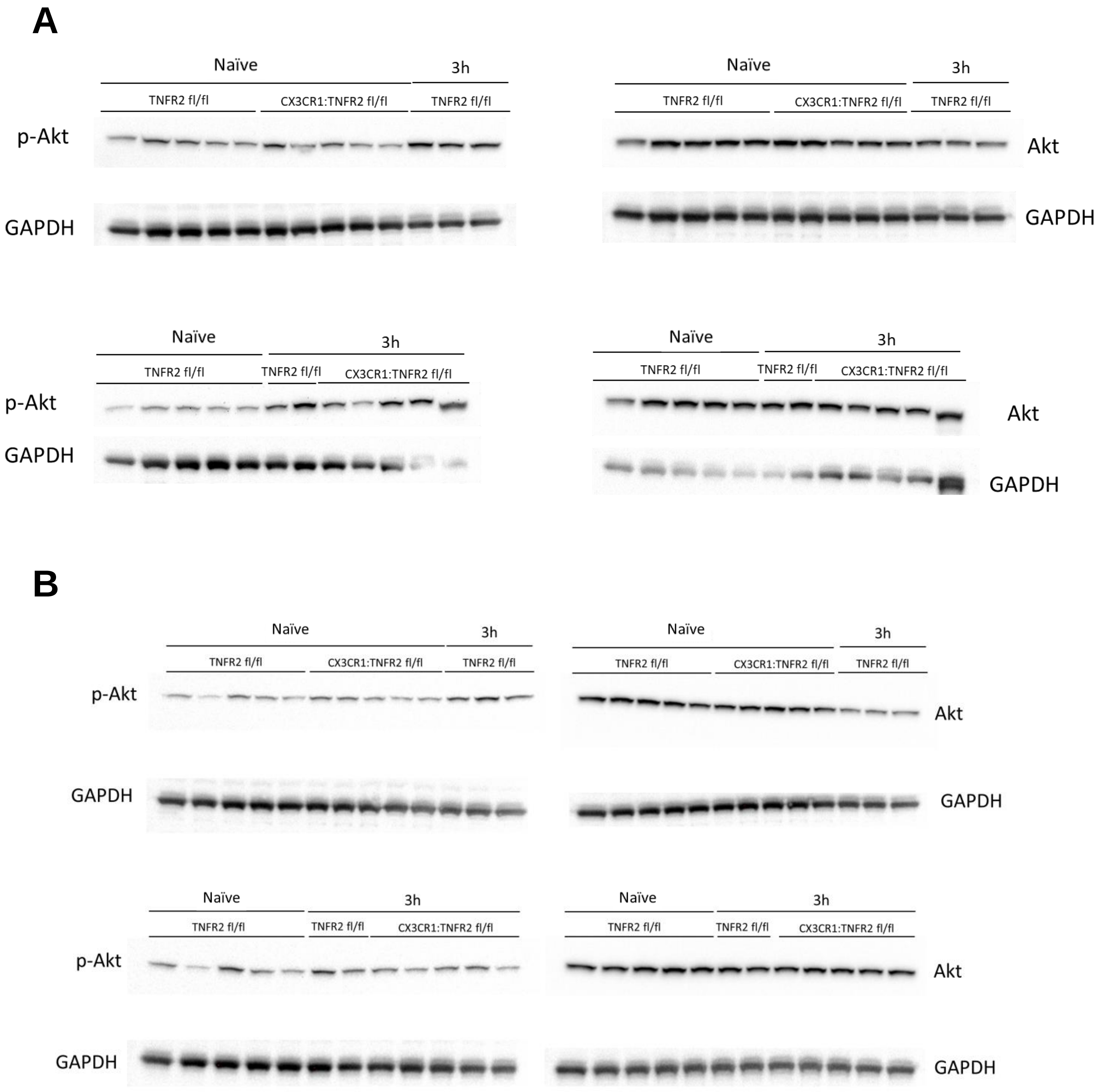

Supplementary Figure 3. (A-B) Images showing the full membranes utilized to quantify by Western blot the protein levels of Akt and phosphorylated Akt (p-Akt) in the brain of female (A) and male (B) *Cx3cr1*^{CreER}:*Tnfrsf1b*$^{\mathrm{fl/fl}}$ mice and *Tnfrsf1b*$^{\mathrm{fl/fl}}$ littermates in naïve conditions and at 3 hours post-MCAo.

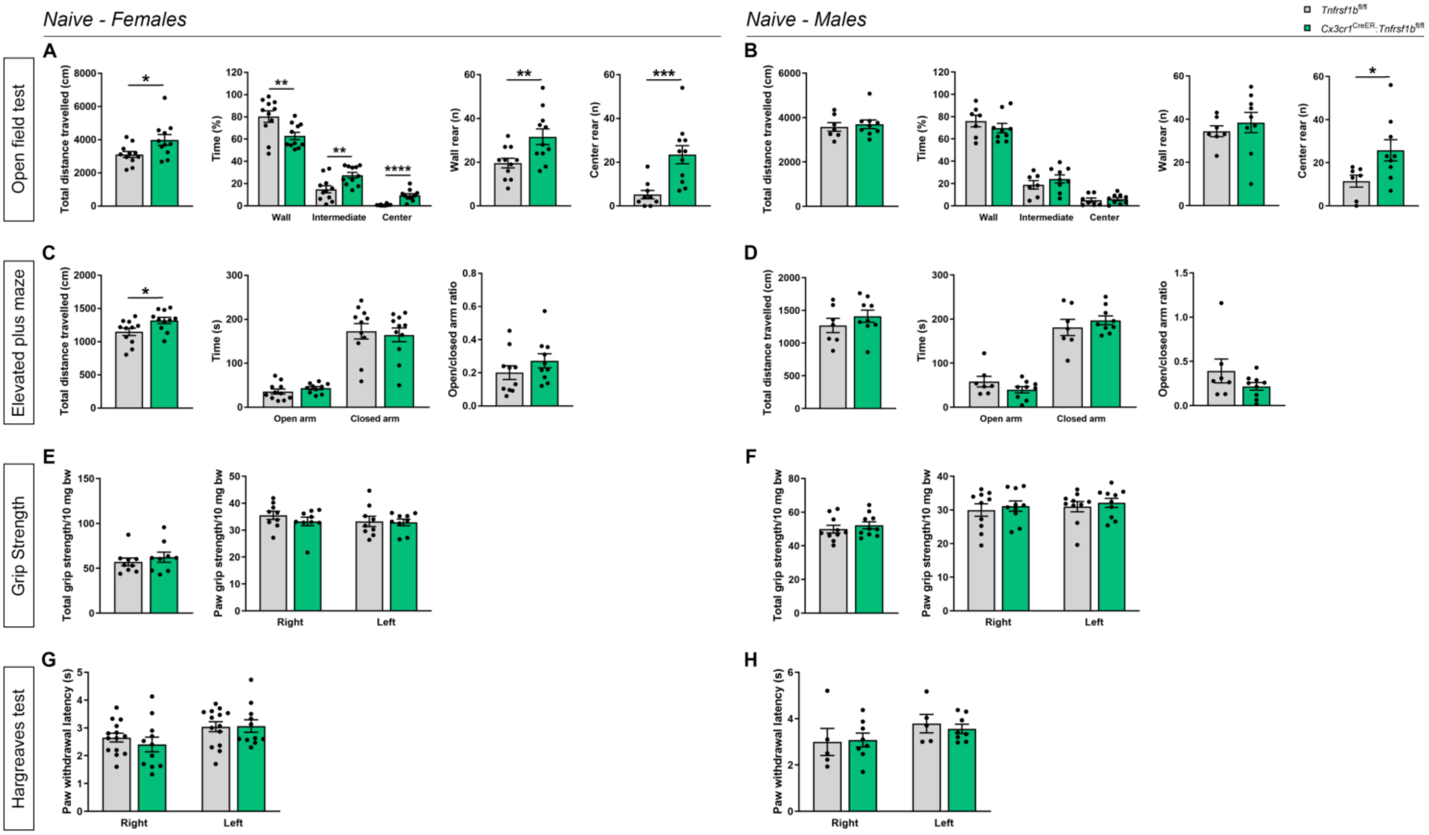

Supplementary Figure 4. (A-B) Quantification of the total distance travelled, time spent in the wall, intermediate, and center regions, wall rearing, and center rearing performed by naïve female (A) and male (B) *Cx3cr1*^{CreER}:*Tnfrsf1b*$^{fl/fl}$ mice and *Tnfrsf1b*$^{fl/fl}$ littermates during the open field test (n=11/group). * p<0.05, ** p<0.01, *** p<0.001; Student's t-test. **(C-D)** Quantification of the total distance travelled, time spent in the open and closed arm, and open/closed arm ratio performed by naïve female (C) and male (D) *Cx3cr1*^{CreER}:*Tnfrsf1b*$^{fl/fl}$ mice and *Tnfrsf1b*$^{fl/fl}$ littermates during the elevated plus maze test (n=11/group). * p<0.05; Student's t-test. (**E-F**) Quantification of the total and paw-specific grip strength of naïve female (E) and male (F) *Cx3cr1*^{CreER}:*Tnfrsf1b*$^{fl/fl}$ mice and *Tnfrsf1b*$^{fl/fl}$ littermates during the grip strength meter test (n=11/group). (**G-H**) Quantification of the right and left paw withdrawal latency of naïve female (G) and male (H) *Cx3cr1*^{CreER}:*Tnfrsf1b*$^{fl/fl}$ mice and *Tnfrsf1b*$^{fl/fl}$ littermates during the Hargreaves test (n=11/group).

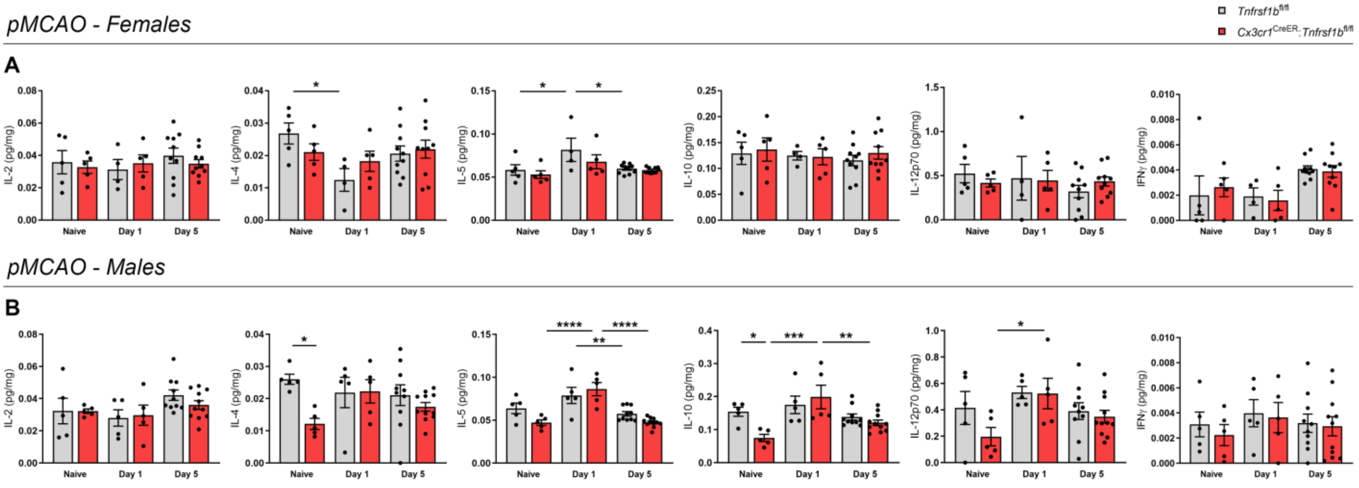

Supplementary Figure 5. (A-B) Quantification of IL-2, IL-4, IL-5, IL-10, IL-12p70, and IFNγ protein levels in the brain of female (A) and male (B) *Cx3cr1*^{CreER}:*Tnfrsf1b*$^{fl/fl}$ mice and *Tnfrsf1b*$^{fl/fl}$ littermates in naïve conditions and at day 1 and 5 post-MCAo (n=5-12/group). * p<0.05, ** p<0.01, *** p<0.001, **** p<0.0001; Two-way ANOVA followed by Bonferroni's multiple comparisons test.

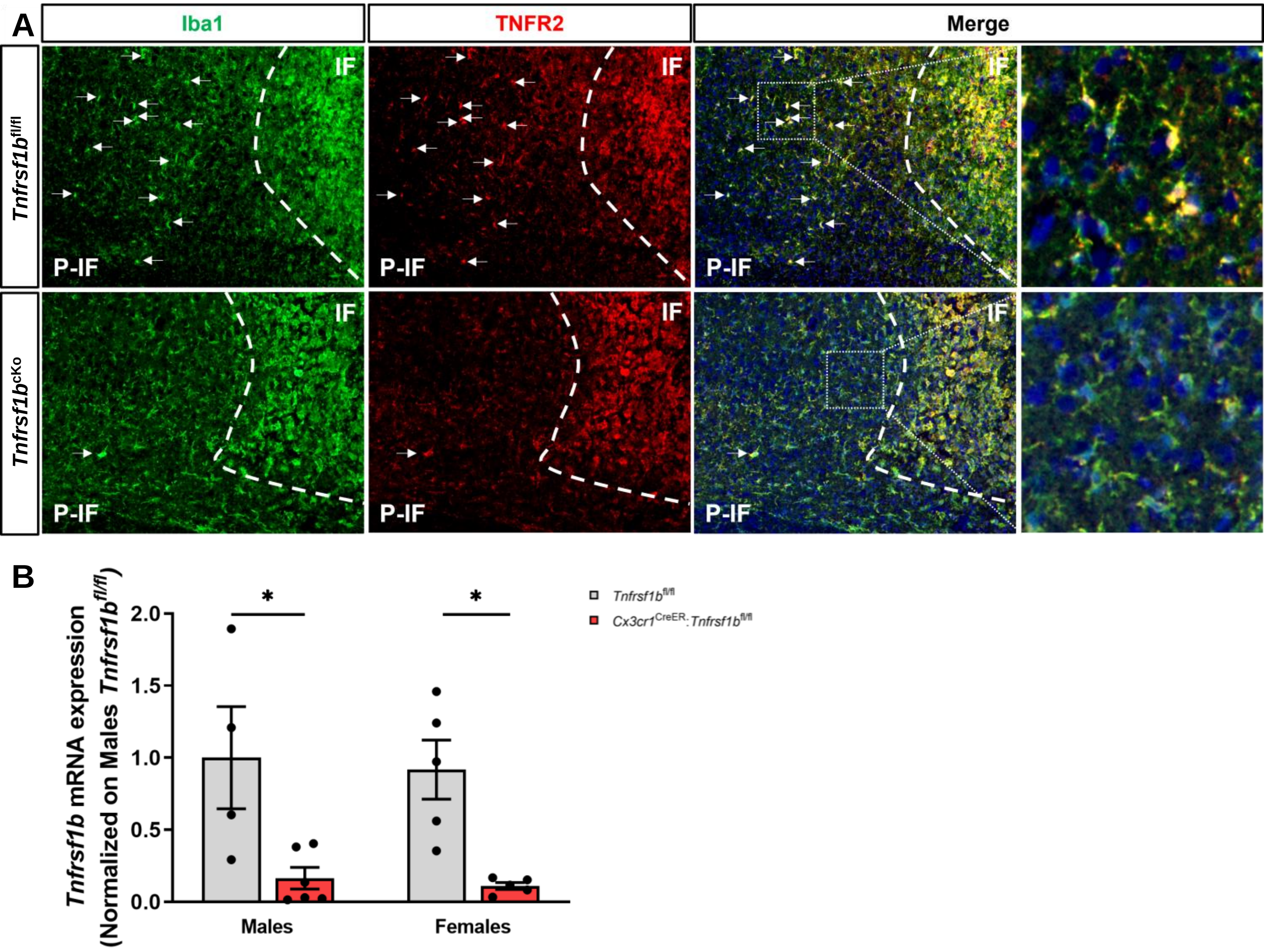

Supplementary Figure 6. (**A**) Representative images of cells stained for Iba1 and TNFR2 in the infarct core (IF) and at the boundary of the ischemic lesion (0-500 µm; peri-infarct region, P-IF) of *Cx3cr1*^{CreER}:*Tnfrsf1b*$^{\mathrm{fl/fl}}$ (*Tnfrsf1b*^{cKo}) mice and *Tnfrsf1b*$^{\mathrm{fl/fl}}$ littermates at day 5 post-pMCAo. Dashed lines separate IF and P-IF. Arrows indicate cells co-expressing Iba1 and TNFR2 in the P-IF. (**B**) Quantification of *Tnfrsf1b* mRNA expression in MACS-sorted CD11b^{+} cells from the ipsilateral cortex of male and female *Cx3cr1*^{CreER}:*Tnfrsf1b*$^{\mathrm{fl/fl}}$ mice and *Tnfrsf1b*$^{\mathrm{fl/fl}}$ littermates at day 5 post-MCAo (n=4-6/group). * $p<0.05$; Two-way ANOVA (Interaction^{ns}, Sex^{ns}, Genotype***) followed by Tukey's multiple comparisons test.
